# Distinct cell state ecosystems for nodular lymphocyte-predominant Hodgkin lymphoma

**DOI:** 10.1038/s41467-025-63339-9

**Published:** 2025-09-26

**Authors:** Ajay Subramanian, Shengqin Su, Jamie Flerlage, Stefan Alig, Sheren Younes, Lianna J. Marks, Chelsea Pinnix, Francisco Vega, Raphael Steiner, Priya Kumar, Heidi Mocikova, Alice Sykorova, Vit Prochazka, Cristiane Milito, Pamela Allen, Darina Paulino, Alan Ramsay, Timothy Flerlage, Monica Palese, Robert West, ChunFang Zhu, Troy Noordenbos, Joseph Schroers-Martin, Shuchun Zhao, Natalie J. Park, Anusha Kalbasi, Everett J. Moding, Aaron M. Newman, Ranjana H. Advani, Richard T. Hoppe, Maximilian Diehn, Yasodha Natkunam, Ash A. Alizadeh, Michael Sargent Binkley

**Affiliations:** 1 Department of Radiation Oncology, Stanford University, Stanford, CA USA; 2 Department of Pediatrics, University of Rochester, Rochester, NY USA; 3 Department of Medicine, Division of Oncology, Stanford University, Stanford, CA USA; 4 Department of Pathology, Stanford University, Stanford, CA USA; 5 Department of Pediatrics, Division of Pediatric Hematology/Oncology, Stanford University, Stanford, CA USA; 6 Department of Radiation Oncology, MD Anderson Cancer Center, Houston, TX USA; 7 Department of Hematopathology, MD Anderson Cancer Center, Houston, TX USA; 8 Department of Lymphoma & Myeloma, MD Anderson Cancer Center, Houston, TX USA; 9 Department of Pathology, St. Jude Children’s Research Hospital, Memphis, TN USA; 10 Department of Hematology, Fakultni nemocnice Kralovske Vinohrady and Third Faculty of Medicine, Charles University, Prague, Czechia; 11 4th Department of Internal Medicine – Hematology, University Hospital and Faculty of Medicine, Hradec Kralove, Czechia; 12 Department of Hemato-oncology, Faculty of Medicine and Dentistry, Palacky University and University Hospital Olomouc, Olomouc, Czechia; 13 Department of Pathology at the Federal University of Rio de Janeiro, Rio de Janeiro, Brazil; 14 Department of Hematology & Oncology Winship Cancer Institute of Emory University, Atlanta, GA USA; 15 University College London Hospitals NHS Foundation Trust, London, UK; 16 Department of Infectious Diseases, University of Rochester, Rochester, NY USA; 17 Department of Biomedical Data Science, Stanford University, Stanford, CA USA

**Keywords:** Hodgkin lymphoma, Cancer microenvironment

## Abstract

Nodular lymphocyte-predominant Hodgkin lymphoma (NLPHL) is a rare cancer, and few studies have comprehensively investigated the immune microenvironment and rare lymphocyte-predominant (LP) cells. Here we develop a NLPHL specific lymphocyte-predominant ecotype (LPE) model to identify 34 distinct cell states across 14 cell types that co-occur within 3 LPEs for 171 cases. LPE1 and LPE2 were characterized by immunosuppressive microenvironments with high expression of *B2M* on LP cells, CD8 T-cell exhaustion, immune checkpoint genes expressed by follicular T-cells, and an improved freedom from progression compared to LPE3 in training (*n* = 109, with 65% LPE1/2) and validation cohorts (*n* = 62, with 61% LPE1/2). We validate the co-occurrence and co-localization of cell states using spatial transcriptomics. Protein expression of HLA-I and HLA-II on LP cells and SSTR2 on dendritic cells was predictive of LPE1 (C-statistic=0.69), LPE2 (C-statistic=0.79), and LPE3 (C-statistic=0.60). This study establishes a clinically relevant biologic categorization for NLPHL.

## Abstract (web summary)

Nodular lymphocyte-predominant Hodgkin lymphoma (NLPHL) is a rare cancer. Here, the authors develop a NLPHL specific model to identify 34 distinct cell states across 14 cell types that co-occur within 3 lymphocyte predominant ecotypes (LPEs) for 171 cases.

## Introduction

Nodular lymphocyte-predominant Hodgkin lymphoma (NLPHL) is an orphan disease which has been challenging to study due to its rarity, representing only 5% of Hodgkin lymphoma (HL) cases^1^. Although age of diagnosis and low tumor cell abundance are common features across all HL subtypes, the malignant lymphocyte-predominant (LP) cells in NLPHL retain their expression of the B-cell receptor and express CD-20 in contrast to classic Hodgkin lymphoma (cHL), prompting consideration of reclassification^2,3^. Further, NLPHL exhibits a unique clinical behavior characterized by indolent lymphoma growth and increased risk for late relapse when compared to cHL^4,5^. The clinical management of NLPHL has largely remained unchanged for many years with few biologic discoveries to aid in the development of new treatments and exclusion from the majority of frontline trials for cHL^5,6^.

Similar to cHL, NLPHL is characterized by an immune cell rich microenvironment with LP cells representing ~1% of tissue specimens^2,7^. Six morphologic subtypes of NLPHL have been described but phenotypic characterization and prognostic significance of these immunoarchitectural patterns (IAPs) is limited^8,9^ and complicated by intra-tumoral IAP heterogeneity^2,9,10^. Despite these limitations, the variant IAPs are associated with advanced clinical stage and risk of transformation to aggressive large cell lymphoma in many studies, and novel techniques to study the microenvironment are needed^9,11–13^. With the advent of single cell sequencing technologies, several large atlases for malignant lymphomas and non-malignant immune cell states have been reported^14–18^, which when combined with bulk RNA-sequencing digital deconvolution algorithms^19,20^, represent powerful tools to comprehensively characterize the microenvironment for a large number of cases.

We utilized a multimodal next-generation sequencing strategy, including bulk RNA-seq, single-cell transcriptomics, spatial transcriptomics, non-invasive genotyping, and spatial proteomics alongside case sharing through the Global nLPHL One Working Group (GLOW)^21^, to characterize the malignant and immune microenvironment phenotypes of NLPHL and assess their prognostic value^14,21–24^. We performed single cell multiplexed fluorescent imaging using CO-Detection by indEXing (CODEX) to generate gold standard cell abundances to benchmark our bioinformatic digital deconvolution methods (see “Methods”)^25^. We performed genotyping from microdissected LP cells in a noninvasive manner via ultrasensitive sequencing of circulating tumor DNA (ctDNA), and characterized cell gene expression state ecosystems (“ecotypes”) which appear to be predictive of outcome^19,20,23,24,26^. We further identify microenvironment features and expressed proteins suitable for immunostaining aiding in future implementation clinically.

## Results

### Patients with newly diagnosed NLPHL

**Fig. 1:**
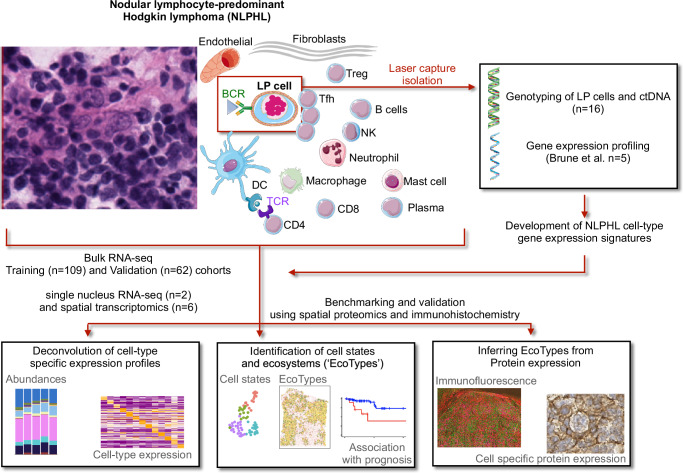
Framework to identify and validate the landscape of cell states and ecosystems for nodular lymphocyte-predominant Hodgkin lymphoma (NLPHL). A schematic displaying the approaches taken to genotype the malignant lymphocyte-predominant (LP) cells, characterize the immune microenvironment, and finally measure B-cell and T-cell receptor diversity within tissue samples. Laser capture microdissection was used to isolate LP cells with subsequent pooling, DNA isolation, and genotyping via targeted hybrid capture assay. Bulk RNA-seq was subsequently performed on patient tissue samples for training (*n* = 109) and validation (*n* = 62) cohorts with digital deconvolution, cell state identification, and ecosystem (EcoTypes) delineated via machine learning algorithms. The cell states were validated for two samples using single nucleus RNA-seq with proximity assessed using spatial transcriptomics for 6 samples. Finally, protein expression of the LP cells may infer LPE.

We assembled a retrospective training cohort (*n* = 109 from 5 institutions) and validation cohort (*n* = 62 from 3 institutions) consisting of patients with newly diagnosed NLPHL with formalin fixed, paraffin embedded tissue available to use for our next generation sequencing analyses. The two cohorts were independent from each other by time collected as well as the institutions being separate in space. Two of the patients had fresh frozen tissue available (Fig. 1). Nearly all tissue specimens were excisional biopsies (*n* = 165, 96.5%) with the remainder consisting of core biopsy specimens (*n* = 6, 3.5%). The tissue diagnosis and IAP were centrally reviewed and scored according to the 5th edition of the World Health Organization classification of Lymphoid Neoplasms^8,27^. Full clinical staging, treatment, and follow-up data were available for 84 patients (77.1%) in the training cohort and 52 patients (83.9%) in the validation cohort (Supplementary Data 1 and 2) with summary statistics provided in Supplementary Data 3. Patients were younger in the training (median, 23 years) versus the validation cohort (median, 50 years, *P* < 0.001). The majority of patients had early stage NLPHL in the training (*n* = 59, 70.2%) and validation cohorts (*n* = 39, 75.0%), in line with the nature of the disease. Additionally, 60 were male in the training (71.4%) and 42 were male in the validation cohort (80.8%). Spleen involvement occurred for 10 (11.9%) and 8 (15.4%) patients in the training and validation cohorts, respectively. Only one death occurred in the training cohort secondary to non-lymphoma causes, and three occurred in the validation cohort with only one secondary to lymphoma.

### Cell type gene expression profiling for NLPHL

**Fig. 2:**
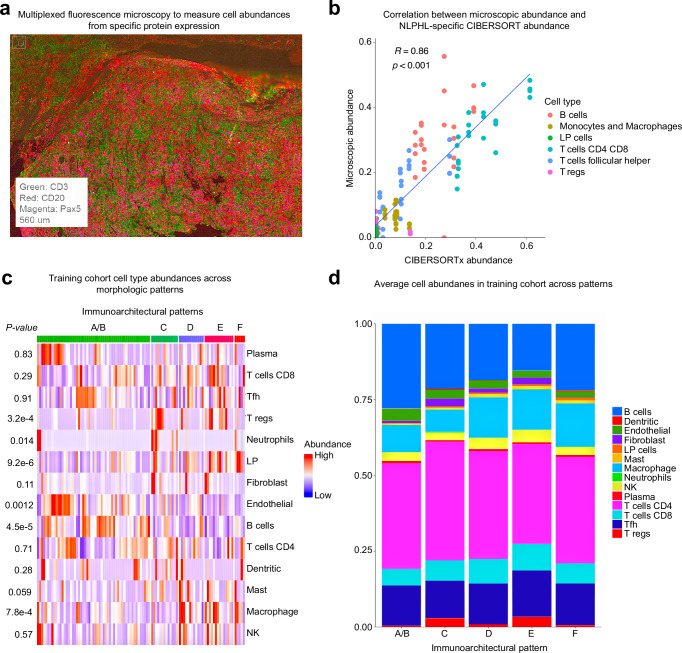
Digital deconvolution of bulk RNA-seq data allows for measurement of cell-type abundance and cell-specific expression. **a** A multiplexed immunofluorescent platform (CODEX) was used to measure cell abundances to serve as a reference to benchmark bioinformatic methods (4 regions of interest per case). **b** Correlation of cell abundances obtained for 6 cases with 4 regions of interest selected per case using CODEX versus values obtained using CIBERSORTx (two-sided Wilcoxon rank sum *P* value and linear regression correlation). **c** Cell abundance as measured by CIBERSORTx^19^ for 14 cell types for each sample in the training cohort (*n* = 109) stratified by immunoarchitectural pattern^8^ with statistical significance of differences across patterns (ANOVA *P* value was calculated to compare abundances across patterns). **d** Average cell abundances measured across immunoarchitectural patterns.

In order to characterize the benign, immune, and malignant cell phenotypes in a high-throughput manner, we developed an NLPHL-specific cell type gene expression signature matrix (Supplementary Data 4), tailored for use with CIBERSORTx, a validated digital bulk RNA-seq deconvolution tool^19^. As described in our Methods, we accomplished this by incorporating gene expression data from micro-dissected LP cells^26^, 22 immune cell types, and other benign cells (epithelial, endothelial, and fibroblast)^19^. After consolidating the immune and benign cell types into the 13 major lineages found most abundantly in lymphomas^20^ and adding LP cells, we obtained a final signature matrix which included 14 total cell types. We scaled down LP cell abundances based on our genotyping results described below. To ensure the robustness of our signature matrix, we took a multifaceted approach to test its accuracy: (1) benchmarking our deconvolved abundances against a gold standard single cell measured abundance profile of overlapping cases using a multiplexed immunofluorescent platform (CODEX, Fig. 2a, b, see “Methods”)^28^, (2) comparison of the performance of our chosen digital deconvolution method versus other established and validated single cell/nucleus approaches (MuSiC^29^, DWLS^30^, and CIBERSORTx using a snRNA-seq derived signature matrix, Supplementary Fig. 1), and (3) investigation of the etiology of LP cell overestimation (see genotyping and snRNA-seq sections below).

We tested the performance of the NLPHL-specific signature matrix using multiple datasets. Importantly, using gene expression data obtained from tonsil (*n* = 4) and plasma depleted whole blood (*n* = 12)^19^, we did not detect any substantial fraction of LP cells suggesting the LP cell type did not overlap with immune cell populations in benign samples (Supplementary Data 5 and 6). Further, we also tested the NLPHL-specific signature matrix using four samples with progressive transformation of germinal centers (PTGC) with nearly undetectable LP abundance (median 0.15%, Supplementary Data 7). 3 of the 4 patients with PTGC had a prior history of lymphoma (2 with prior NLPHL) without known recurrence. Of note, 1 of 4 PTGC samples with the highest LP abundance (0.2%) occurred in a patient who eventually developed clinical recurrence of NLPHL following the biopsy.

Subsequently, we performed bulk RNA-seq (Supplementary Data 8) and measured the abundance of the 14 cell types in our training cohort using CIBERSORTx. Notably, we observed very similar relative abundances across IAPs as reported by pathology studies further affirming the accuracy of the results obtained through digital deconvolution (Fig. 2d)^2,8,9,28^. We specifically saw significantly higher abundance of Treg cells, LP cells, monocytes/macrophages, and a lower abundance of B cells in IAP patterns C-F (Fig. 2c, d). We similarly performed bulk RNA-seq on the validation cohort (Supplementary Data 9). To further evaluate the ability to measure rare cell abundances, we also observed significant modest correlation between rare immune cell abundances such as mast cells obtained via CIBERSORTx versus the abundance measured via immunostaining for MCT protein (Supplementary Fig. 2).

### Genotyping NLPHL from tissue and plasma

**Fig. 3:**
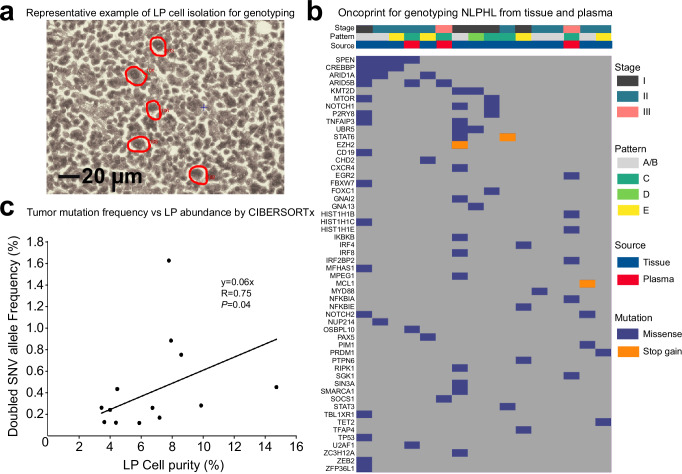
Genotyping of LP cells from cellular DNA using laser capture microdissection and circulating tumor DNA. **a** A representative NLPHL tissue sample with the LP cells outlined in red which were subsequently microdissected. Approximately 200 LP cells per tissue sample were isolated and pooled for DNA isolation, library preparation and sequencing. **b** Oncoprint demonstrates the recurrent somatic tumor mutations enriched in microdissected tissue or ctDNA as compared to the allele frequency found when performing bulk DNA-seq. **c** Scatterplot shows strong correlation between 2*single nucleotide variant allele frequency value versus LP cell abundance as measured via CIBERSORTx (linear regression correlation and two-sided *P* value calculated by Wilcoxon rank sum).

Genotyping NLPHL from tissue has proven to be very challenging due to the paucity of LP cells and the presence of non-specific cell surface proteins shared by the abundant B-cells^2,31,32^. To overcome this challenge, we performed laser capture microdissection of LP cells (Fig. 3a) from 15 cases with subsequent pooling of LP cells for each patient, isolation of DNA, and preparation of sequencing libraries using a hybrid capture assay with a targeted panel of 151 genes reported as recurrently mutated in B-cell lymphomas (Fig. 3b)^23,24,33^. For 13 of 15 cases with sufficient tissue, we also performed bulk sequencing and genotyping using the same hybrid capture assay. Given the lower sequencing depth obtained from the micro-dissected cases (median = 114X, range = 12–1016X, Supplementary Data 10), after filtering to guard against calling clonal hematopoiesis variants (see “Methods”), we conservatively only called somatic mutations that were concordant between the micro-dissected and bulk sequencing results with germline removal of SNPs. One sample had no identified somatic tumor mutations after filtering and another did not have bulk DNA sequencing available as the tissue block had been exhausted. Finally, we were also able to successfully isolate DNA from plasma and perform genotyping for 6 patients from plasma (Fig. 3b) but were unsuccessful in identifying any somatic tumor mutations in 3 of 4 early stage cases. We attempted to look for gene fusions using the bulk RNA-seq data for the 4 patients without detectable somatic tumor mutations (3 profiled from cell free DNA, 1 from FFPE tissue) but did not find any fusion events^34^. Examining the spectrum of mutations (Fig. 3b), nearly half of the cases (7 out of 16, 44%) had mutations in epigenetic genes (*CREBBP/KMT2D/EZH2*) and 6 out of 16 cases (31%) had mutations in the NOTCH signaling pathway (*SPEN/NOTCH1/NOTCH2/FBXW7*).

For the 13 samples where somatic tumor mutations were identified and where bulk sequencing data were available, we conducted a comparison between the digital cytometry-derived abundance of LP cells (obtained via CIBERSORTx) and twice the value of the median somatic nucleotide variant allele frequency (representing likely clonal mutations). This comparison revealed a modest positive correlation (*R* = 0.75, Fig. 3c). As we would expect a 1:1 ratio between the LP cell abundance and twice the somatic tumor mutation frequency in bulk tissue, we scaled down the LP abundance obtained from CIBERSORTx by 16.67 representing one divided by the slope of the linear fit shown in Fig. 3c for subsequent analyses. This decrease in LP cell abundance was accompanied by a symmetric increase in the abundance of the other cell types.

### Identification and validation of cell states

**Fig. 4:**
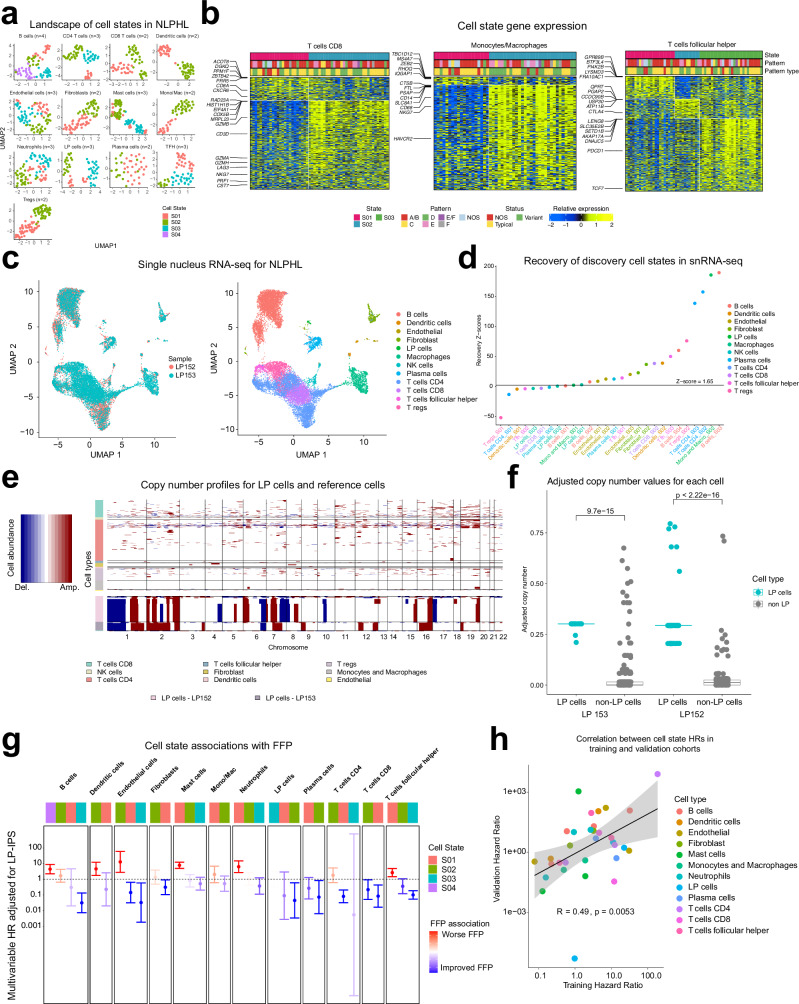
Identification of cell states within the training cohort. **a** Using the machine learning algorithm EcoTyper, UMAPs show a range of 2–4 cell states observed for LP and 13 immune cell types resulting in a total of 34 cell states. **b** Gene expression heatmaps demonstrate characteristic gene expression profiles for cell states observed for CD8 T-cells, Macophages, and Tfh cells. **c** UMAPs demonstrate the representation of isolated nuclei (left) and the assigned cell types obtained from snRNA-seq (right). **d** Nearly all cell states from NLPHL EcoType 2 or in high abundance within two snRNA-seq samples are significantly recovered (permutation testing yielded z scores which were combined into a meta z score using Stouffer’s method where z scores > 1.65 [one sided *P* < 0.05] were deemed significant). **e** Copy number alterations are more frequent for LP versus immune cell types across the genome. **f** Scatter box plot shows increased copy number alterations versus benign cells. (two-sided Wilcoxon rank sum *P* values and box plot shows the median along with the interquartile range overlayed with the dot plot of all values) **g** Cell state associations adjusted by the LP-IPS for samples in the training cohort (*n* = 109) (multivariable Cox proportional hazards models adjusted for LP-IPS with error bars representing 95% confidence intervals). **h** The hazard ratios obtained for the validation cohort (*n* = 62) significantly correlate with the values obtained for the training cohort (**g**) (multivariable Cox proportional hazards models adjusted for LP-IPS were used along with Spearman correlation, shaded standard error bands, and two-sided Wilcoxon rank sum *P* values).

We then employed the EcoTyper machine learning framework^20^ to identify transcriptional cell states for each cell type obtained from CIBERSORTx for the training cohort. EcoTyper uses non-negative matrix factorization (NMF) to identify transcriptionally distinct cell states independent of morphologic IAPs which score tissue architecture. We identified a range of 2–4 cell states (S01-S04) for the 14 cell types for a total of 34 distinct cell states (Fig. 4a and Supplementary Data 11–23).

For annotation, we compared the state defining expressed genes to single cell RNA-seq atlases for DLBCL and immune cells^15^, as there is no available atlas for NLPHL. Comparing our CD4 cell states to the DLBCL atlas^15^, S01 had overlap with naïve CD4 expression (*LUC7L3, LDLRAP1, TCF25, TNRC6B, AQP3*), S02 had overlap with naïve clusters 3–5 (*ICOS, INTS6, HSPH1, SRSF2, CD55)*, and S03 had overlap with naïve cluster 11 (*PRKX, TCF7, CARS, NELL2*) (Supplementary Data 20). Looking at CD8 cells and comparing to the DLBCL atlas^15^, we observed a high expression of genes from the exhausted T-cell clusters 13 and 14 for S02 (*CCL5, GZMB, LAG3, CD8B*) versus S01 which had high expression of CD8A associated with non-MHC-restricted cytotoxicity (Fig. 4b)^35^. For macrophages, we used a myeloid cell atlas^16^ finding both S01 (*MS4A7*) and S02 (*CTSB, CD68, CD163*) had classic M2 markers expressed, although S02 had a *C1QC* “phagocytosis-like” phenotype (Fig. 4b). Interestingly, macrophage S01 had increased expression of *MYD88*^36^. For follicular helper T-cells (Tfh), S01 had high expression of T-cell activation genes implicated in anti-tumor immunity including *PI4K2B*^37^ as well as the ganglioside checkpoint gene *ST8SIA1*^38^, S02 had increased CTLA-4 expression, and S03 had increased expression of PD-1 and some overlap with exhausted cluster 13 (*TNFRSF18, DUSP4*) (Fig. 4b). For B-cells, S01, S03, and S04 had plasmablast phenotypes while S02 had a dark zone phenotype (Supplementary Table 11)^39^. Both regulatory T-cell (Treg) states had expression of effector genes^40^, but Treg S02 was characterized by high *CD27* expression compared to S01 which more highly expressed *CD7*, both markers of T cell exhaustion (Supplementary Table 22)^41,42^. Finally, we were intrigued to identify 3 cell states for LP cells where LP states 01 and 02 had significantly higher expression of B2M versus LP03 (*P* < 0.05, Supplementary Fig. 3), similar to findings reported for Reed Sternberg cells^43^.

To further validate our cell states, we performed single nucleus RNA-sequencing (snRNA-seq) on two fresh frozen tissues. After filtering (see “Methods”), we recovered 17,303 nuclei (7399 from sample 1 and 9904 from sample 2). We achieved excellent resolution of our cell types, including the rare LP cells, as shown in the UMAP with nearly equal distribution and representation by both samples (Fig. 4c). There was a significant correlation between relative cell abundances obtained via snRNA-seq versus CIBERSORTx from bulk RNA-seq (*R* = 0.75, *P* < 0.001, Supplementary Fig. 4). These samples were also characterized with spatial transcriptomics (see below) with a range of LPE3 abundance. Additionally, we successfully recovered these co-occurring cell states with the exception of Treg S01 as only one sample had measurable Treg abundance assigned to Treg S02 (Fig. 4d). Using 11 benign cell types as reference, we also applied inferCNV^44^ to the snRNA-seq data which highlighted that the LP cell cluster had significantly higher amplifications and deletions compared to immune cells further supporting its malignant phenotypic assignment and higher estimated LP abundance prior to our corrective scaling of cell types (Fig. 4e, f). Additionally, we found amplification in *CD274 (PD-L1)* for the LP nuclei from one sample relative to immune nuclei, similar to that reported for cHL (Supplementary Fig. 5)^45^. As another explanation for the higher estimated LP abundance prior to corrective scaling (see signature matrix generation), we observed LP cells had significantly higher RNA feature count (unique genes) and n count (total number of RNA molecules per cell) suggesting RNA content was also higher for LP cells versus others (Supplementary Fig. 6).

Finally, we aimed to evaluate the prognostic impact associated with each individual cell state in the training cohort. We performed individual multivariable Cox regression analyses for each cell state adjusted for the lymphocyte-predominant international prognostic score (LP-IPS). These analyses revealed that the LP S02 was associated with improved freedom from progression (FFP) (Fig. 4g). Concordant results were observed when performing the same Cox regression analyses adjusted for LP-IPS in the validation cohort (Fig. 4h). For these survival analyses, we excluded patients who received rituximab alone, as this treatment is considered palliative with inferior outcomes compared to definitive treatment options^46^.

### Cell state ecosystems for NLPHL

**Fig. 5:**
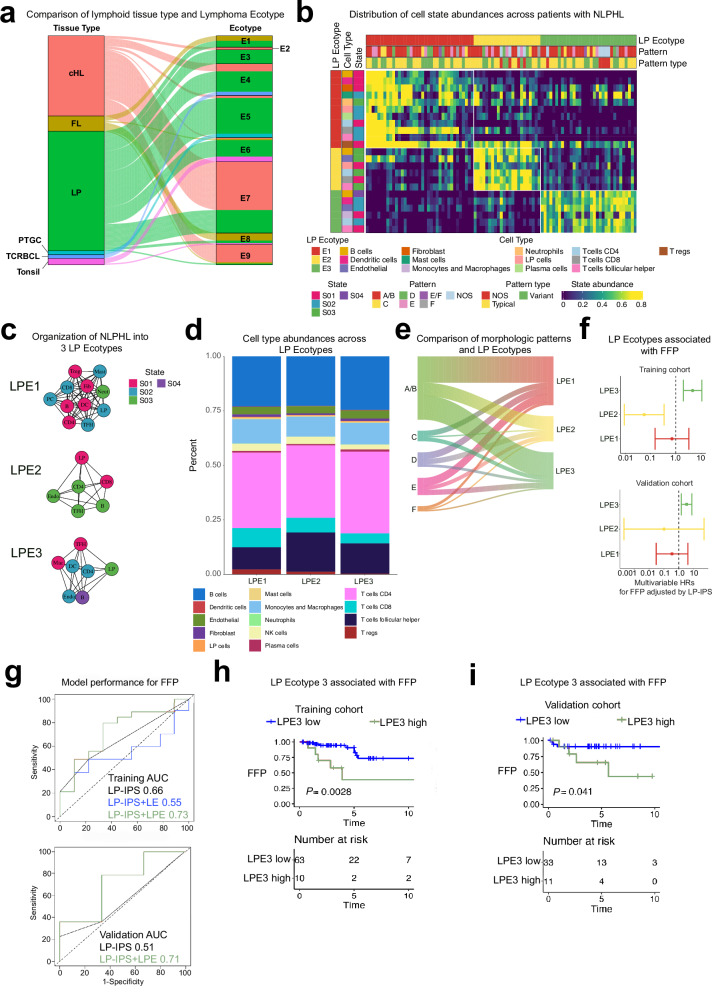
Characterization of cell state ecotypes and association with freedom from progression. **a** Only a minority of NLPHL samples have microenvironmental cell states that overlap with other B-cell lymphomas and lymphoid proliferations as defined by Lymphoma EcoTyper^20^ (thickness of band represents percent of samples). **b** Heatmap demonstrates abundance of cell state phenotypes and clustering into three lymphocyte-predominant ecotypes (LPE). **c** Network plots demonstrate the ecotype defining cell states for the LPE model (Jaccard index values were calculated for the length of the edges). **d** Abundance of cell types stratified by LPE. **e** Riverplot shows a fairly uniform distribution of immunoarchitectural patterns represented within each LPE (thickness of band represents percent of patients within each category). **f** Multivariable Cox regression models adjusted for the LP-IPS show LPE3 is associated with significantly worse freedom from progressive lymphoma following definitive treatment in the training (top) and validation (bottom) cohorts. (multivariable Cox regression models adjusted for LP-IPS with error bars represented as 95% confidence intervals) **g** Receiver-operator-characteristics curves show LPE3 abundance improves prediction of relapse by 7% and 16% for the training (top) and validation cohorts (bottom), respectively (area under the curve values shown). Kaplan-Meier curves demonstrate patients with high LPE3 classification have worse freedom from progressive lymphoma after definitive treatment in the training (**h**) and validation (**i**) cohorts (two sided *P* values are calculated by log-rank test).

We next used EcoTyper to determine co-occurring cell state ecosystems. Initially, we first ran the published Lymphoma EcoTyper model in recovery mode to evaluate its performance for the B-cell lymphoma model on NLPHL which is a B-cell malignancy^20^. Given the controversy regarding the classification of NLPHL, we included several other sample types along with our training NLPHL cohort: newly diagnosed cHL (*n* = 65), T-cell/histiocyte rich large B-cell lymphoma (TCRBCL [*n* = 5]), follicular lymphoma (*n* = 13), PTGC (*n* = 4), and tonsil (*n* = 5) (Supplementary Fig. 7). As shown in Fig. 5a, we were surprised to observe that only a minority of NLPHL samples showed overlap with TCRBCL, cHL, and FL, indicating the majority of NLPHL possess a microenvironment distinct from other B-cell lymphomas. Moreover, 5 of 9 Lymphoma EcoTypes included >10 NLPHL samples indicating either microenvironment heterogeneity in NLPHL or a non-optimal clustering performance of the Lymphoma EcoTyper model.

To align with the objectives of our study, which included incorporating malignant LP cells as well as assessing the clinical utility of microenvironmental features, we reran the EcoTyper algorithm in discovery mode to develop a specific NLPHL EcoTyper (“lymphocyte-predominant” EcoTyper, [LPE]). We identified 3 distinct LPEs (Fig. 5b, 39.8% LPE1, 24.7% LPE2, and 35.5% LPE3 for 93 assigned patients) with co-occurring cell states shown in the network plots in Fig. 5c. There was no statistical difference in age, gender, or LP-IPS across ecotypes. We observed significantly increased abundance of CD8 T-cells and Tregs for LPE1 and increased abundance of Tfh cells for LPE2 and LPE3 without other significant differences in cell abundances across the LPEs (Fig. 5d). Notably, IAP subtypes were equally distributed across all LPEs, including variant E (Fig. 5e). We then applied our LPE model to our validation cohort and obtained a very similar distribution of the IAPs across the LPEs suggesting the presence of the co-occurring cell states was independent of morphology (Supplementary Fig. 8). After adjusting for the LP-IPS in a multivariable model, we observed increasing abundance of LPE3 was associated with worse FFP in the discovery and validation cohorts with LPE3 representing 35.5% and 39.3% of the cohorts, respectively (Fig. 5f).

The LPE3 abundance (expressed as a continuous variable) improved the predictive performance of the LP-IPS model as evidenced by ROC analysis (Fig. 5g, AUC = 0.66 for LP-IPS versus 0.73 for LP-IPS + LPE3). Our model outperformed LP-IPS+Lymphoma EcoType 5 (the most abundant ecotype in the B-cell model^20^ with AUC = 0.55). A similar improvement was observed in the validation cohort (Fig. 5g, AUC = 0.51 for LP-IPS versus 0.71 for LP-IPS + LPE3). Finally, using a threshold optimized on the log-rank statistic for FFP, a high abundance of LPE3 was significantly associated with worse FFP in the discovery (Fig. 5h) and validation cohorts (Fig. 5i) by Kaplan-Meier analysis.

Although limited by small patient numbers, we were also intrigued to see that in the training cohort, patients with LPE3 assignment receiving single modality treatment (surgery/radiotherapy/chemotherapy) had significantly worse FFP compared to those with LPE1 and LPE2 (Supplementary Fig. 9a) with concordant numeric results observed for the validation cohort (Supplementary Fig. 9b). However, based on prior large clinical studies, patients receiving combined modality therapy have excellent outcomes with very few recurrences^13^. Indeed there was no longer a FFP difference for the small subgroup of patients receiving combined modality treatment with chemotherapy and radiotherapy in the training and validation cohorts, suggesting LPE3 may be predictive of worse outcome after single modality but not combined modality therapy (Supplementary Fig. 9c, d).

### Spatial distribution of cell states and ecotypes

**Fig. 6:**
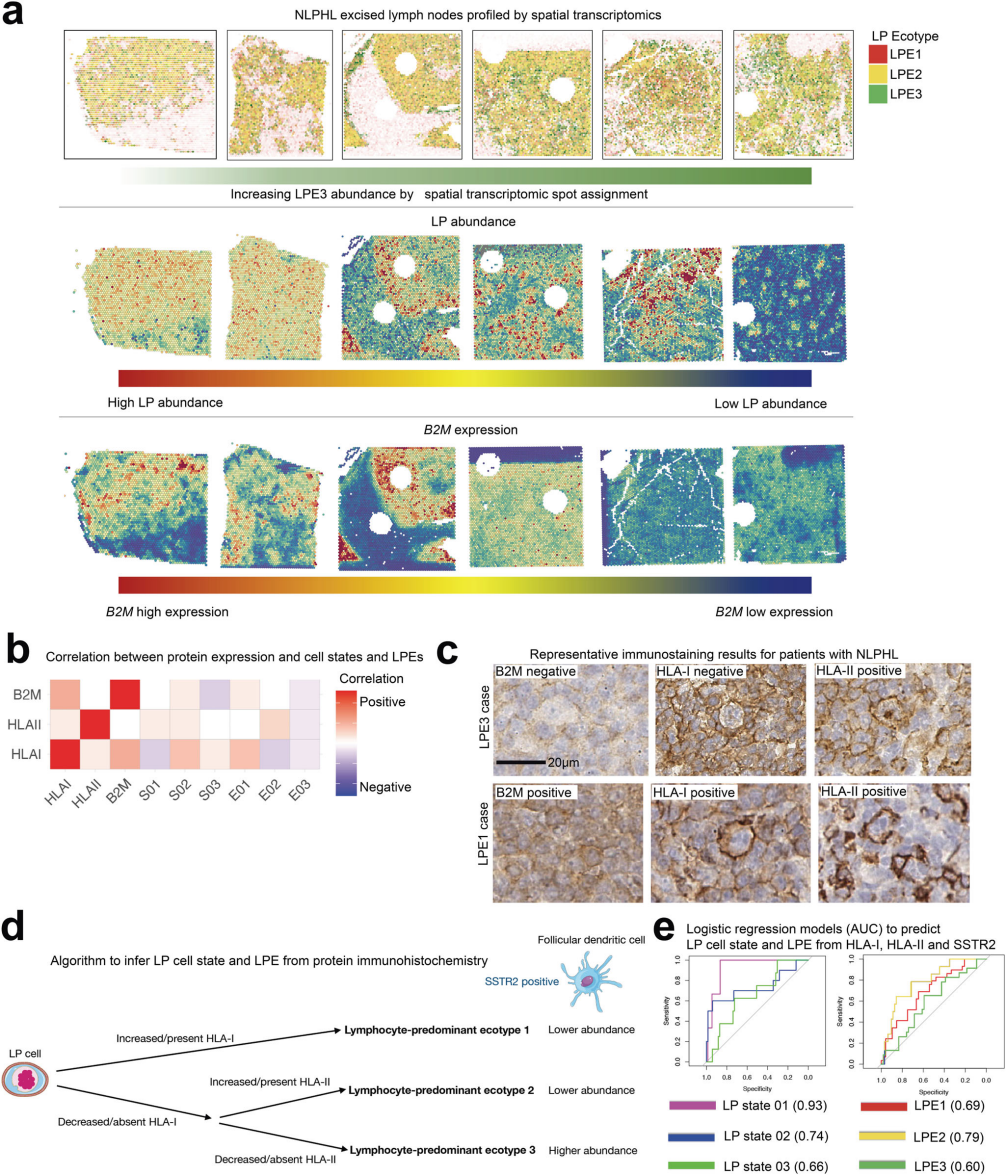
Spatial validation of cell states and cellular proximity. **a** Spatial transcriptomics via the Visium platfrom (10X genomics) demonstrates LPE abundance per spot with increasing LPE3 abundance moving from left to right along the top panel. LP abundance per spot are shown in the middle row. B2M expression per spot is shown on the bottom row. **b** Correlation matrix between immunohistochemical antibody markers and LP cell states and lymphocyte-predominant ecotypes. **c** Representative examples for patients with NLPHL with LP cell negativity and positivity for B2M for the top (LPE3) and bottom (LPE1) panels respectively. **d** Schematic algorithm demonstrating the use of protein expression markers to infer LPE. **e** Logistic regression models to predict LP cell states and LP ecotype 3 using protein expression for HLA-I, and HLA-II on LP cells and SSTR2 on dendritic cells (area under the curve values displayed next to LP states and LPEs).

Utilizing the co-occurring cell state LPEs as a framework, we next used spatial transcriptomics to assess cell state proximity and to validate dispersion of the cell states and ecotypes present. For this analysis, we used six tissue specimens, one from a patient with both pattern A and D IAPs, and four others from separate patients, each with a single, pure IAP (Fig. 6a). Although there was a spectrum of samples ranging from low to high LPE3 abundance spots (Fig. 6a), we did verify significant dispersion of the cell states of all 3 LPEs using Moran’s I for all samples (*P* < 0.05). Additionally, there was a higher number of LP-rich spots in cases with low LPE3 abundance expressing *B2M (P* = 2.2e-16, Fig. 6a*)*. Finally, higher LPE3 abundance in bulk RNA-seq correlated with higher abundances of LPE3 in the visium data (*r* = 0.96, *P* = 0.0024).

To further validate our findings and identify immunostains that may infer LPE, we performed immunostaining for B2M, HLA-I and HLA-II, including all samples with remaining tissue from the discovery and validation cohorts (*n* = 121). As B2M is a component of HLA class I, we observed an expected significant correlation between increasing B2M protein expression and HLA-I expression (*r* = 0.4, *P* = 0.003, Fig. 6b). Similar to findings in cHL^43^, there was no significant correlation between B2M and HLA-II or HLA-I and HLA-II (*P* > 0.05 for both comparisons, Fig. 6b). Representative examples are shown in Fig. 6c and Supplementary Fig. 10 (B2M positive) and Supplementary Fig. 11 (B2M negative). Given the collinearity of B2M and HLA-I, we did not include B2M in our protein expression model. To serve as a microenvironmental positively correlated with LPE3, we used SSTR2 to stain for follicular dendritic cells which were more abundant in LPE3.

We set out to develop a multivariable logistic regression model including HLA-I, HLA-II, and SSTR2 to infer LP cell state and overall LPE. As summarized in Fig. 6d, positive protein expression of HLA-I was significantly associated with LPE1, HLA-II was significantly associated with LPE2, and SSTR2 positive dendritic cells were positively associated with LPE3. The predictive performance of this multivariable logistic regression model is summarized in Fig. 6e, and these three proteins predicted LPE1 (AUC = 0.69), LPE2 (AUC = 0.79), and LPE3 (AUC = 0.60). Thus, it appears immunostaining may serve as an indicative tool to infer high LPE abundance in tissue samples from patients with NLPHL.

### Reconstruction of T-cell and B-cell receptor repertoire from RNA-seq data

**Fig. 7:**
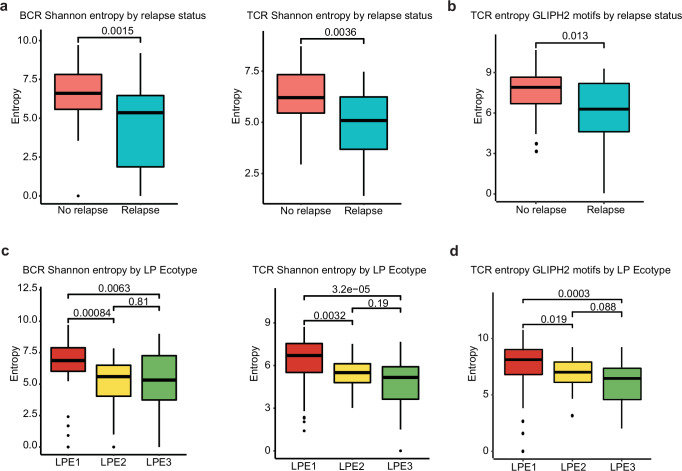
Reconstruction of B-cell and T-cell receptor sequences from RNA-seq data with assessment of diversity across ecotypes and relapse cases. **a** Diversity measured by Shannon entropy for BCR and TCR reads were significantly lower in relapsed versus no-relapse samples. **b** Similarly, there was a reduced diversity of unique TCR motifs by Shannon entropy for relapse versus no relapse samples using the GLIPH2 algorithm^48^. **c** Diversity of BCR and TCR reads by Shannon entropy were significantly lower in LPE2 and LPE3 versus LPE1. **d** Similarly, the diversity of unique motifs per sample was lower for LPE3 and LPE2 versus LPE1 using GLIPH2. For (**a**–**d**), the middle line represents the median value and IQR for the boxplot with whiskers determined by Tukey method. The *P* values are obtained from two-sided Wilcoxon rank-sum tests.

As a final objective, we were interested to determine if T-cell (TCR) and B-cell receptor (BCR) repertoire of the immune microenvironment differed between relapse and no-relapse cases as well as across different LPEs. Using the TRUST4 algorithm applied to the training cohort^47^, we observed relapse cases had lower diversity using Shannon entropy for both TCR and BCR repertoires as compared to no-relapse cases (Fig. 7a). These findings were concordant to results obtained using GLIPH2^48^ which showed lower Shannon entropy values in TCR motifs for relapsed (Fig. 7b). We also found LPE1 had significantly higher TCR and BCR repertoires as measured by Shannon entropy compared to LPE2 and LPE3 cases when using TRUST4 (Fig. 7c) and higher TCR unique clusters using GLIPH2 (Fig. 7d). Although we expected LP cells were of too low abundance in the bulk RNA-seq data to have reliably detected BCR, we did observe some cases had BCR with associated antigens for *Moraxella* (*n* = 8, 7.3%) and *Rothia* (*n* = 7, 6.4%) spp. as reported recently^49^. This finding suggests that even in the context of low-abundance cell types, meaningful insights can be gleaned about the receptor repertoire and its potential antigenic interactions.

## Discussion

In the most comprehensive study to date focusing on the NLPHL microenvironment, we used a multimodal approach to characterize the malignant and immune cell states. We identified 3 LPEs demonstrating LPE3 may be predictive for worse FFP after single modality but not for combined modality therapy. Our LPE model improves the prediction of relapse by 7–20% over that obtained for the LP-IPS alone in our training and validation cohorts and, beyond risk stratification, offers new insight into the LP immune evasion and surrounding immune cell milieu. The concordance of our LPE incidences and prognostic implications between the training and validation cohorts is reassuring as patients were older with a slightly higher percentage of advanced stage cases in the validation cohort. As treatment associated deaths outweigh lymphoma-specific deaths after conventional therapy^5^, it’s highly pertinent to note more than 60% of patients in our training and validation cohorts with LPE1 and LPE2 have excellent outcomes with evidence of T-cell exhaustion and checkpoint gene expression which may be suitable for future preclinical studies evaluating the use of immune checkpoint inhibitors^50^. Although we adjusted for management type in our regression analyses, the training and validation cohorts represent a heterogeneous group of patients managed with a variety of approaches, and it is probable that the LPE model will have higher performance in cohorts of patients who had more uniform treatments. Overall, our LPE classification enriches our understanding of the microenvironment and provides a clinically relevant leap forward in our understanding of NLPHL biology beyond the 6 morphologic IAPs that were not associated with PFS or OS in a large multi-center study after adjustment for LP-IPS factors^13^.

The classification of NLPHL has become increasingly controversial with proposals for reclassification despite there being very scant supporting biologic evidence^3^. To address this, we profiled the LP cell via genotyping and gene expression assays along with characterization of the microenvironment. Despite having low depth in the sequencing of LP cells, we did observe some recurrent mutations in epigenetic genes as well as NOTCH signaling pathway genes. We guarded against calling clonal hematopoiesis variants through germline comparison and focusing on missense and nonsense mutations. Our results have some overlap in somatic tumor mutations of *CREBBP, EP300, SOCS1* as reported by another small series of 17 patients with NLPHL that performed genotyping using a 61-gene panel^31^. Given the laborious nature of microdissection and low sequencing depth, a more efficient genotyping assay for NLPHL is crucial. Unfortunately, based on our small number of samples, it appears genotyping via ctDNA^24^ may not be viable for early stage samples likely due to low DNA shedding. However, surveillance of advanced stage NLPHL via ctDNA may be an attractive option to monitor tumor burden and possibly transformation^51^. Despite some patients with NLPHL transforming and containing overlapping mutations with TCRBCL^31^, based on our cell state and EcoType characterizations, NLPHL has a unique microenvironment with only a small minority of cases sharing cell states with TCRBCL or FL and a larger overlap with cHL.

Having established the ability to resolve cell types and states for NLPHL, we characterized 34 distinct cell states across 14 cell types and 3 co-occurring EcoTypes. LPE1 and LPE2, representing approximately 60% of cases, had evidence of T-cell exhaustion similar to DLBCL^15^ and T-cell expression of checkpoint genes. Similar to DLBCL, the macrophage state expressing *CD68* appears to be associated with a better prognosis for NLPHL^52^. LPE1 had CD8 cells expressing *LAG3* similar to findings reported for cHL^14^, but in contrast MHCII protein expression was higher in our cohort for LPE1 high abundance cases. Also similar to cHL^43^, the LP state 2 associated with LPE1 had higher expression of *B2M* and was also associated with a favorable prognosis. We were intrigued that despite the large heterogeneity observed for DLBCL^15^, we only identified 3 LP cell states.

Finally, we reconstructed the TCR and BCR repertoires from RNA-seq data finding relapse cases had lower TCR diversity similar to findings for cHL^53^. Additionally, LPE3 had lower diversity of TCR and BCR compared to LPE1. Given reported favorable responses to immune checkpoint inhibitors with high TCR diversity, in addition to the microenvironment cell state gene expression profiles, LPE1 + LPE2 may respond more favorably to immunotherapy compared to LPE3 but additional preclinical studies are required. Although we did detect antigens originating from Rothia and Moraxella spp. as reported in the literature^49^, since our data was bulk RNA-seq, it likely did not contain sufficient quantities of RNA from LP cells to allow for comprehensive profiling of the BCR found on the LP cell. Future single cell and nucleus RNA-seq studies may allow for further discovery of the expressed BCR on the malignant LP cells to aid in a better understanding of possible inciting bacterial exposures.

In summary, we have identified distinct cell states for NLPHL that will fuel de-intensification approaches for the majority of patients who have LPE1 and LPE2 cell state phenotypes. Routine immunohistochemistry markers such as antibodies for SSTR2, HLA-I, and HLA-II may infer LPE type. Finally, more intensive treatment such as combined modality therapy may be warranted for patients with LPE3 phenotype.

## Methods

### Human subject and sample collection

We performed a multi-center retrospective study of 171 patients diagnosed with NLPHL at 8 different institutions. The training cohort consisted of cases from 5 institutions, and the validation cohort contained cases from 3 institutions. Institutional review board approval was obtained at all institutions. Data and materials transfer agreements were obtained to provide central review of all materials as well as genomic analyses. When clinical data was available, we collected baseline clinical information, treatments received, and details regarding follow up.

### Central pathology review and IAP scoring

All cases were centrally reviewed by a fellowship trained hematopathologist (YN). All IAPs were scored, and the presence of any variant pattern was scored even if minor. For analysis, samples with composite IAPs were grouped with the higher clinical risk pattern as per a large multi-institutional analysis^54^. Mast cells immunohistochemical staining was performed on a tissue microarray containing a large percentage of our samples for Mast Cell Tryptase (MCT, clone G3 mouse, MAB1222 (Millipore); dilution 1:8000 performed on Roche/Ventana Ultra automated immunostainer) with estimation of mast cell abundance using quantification by Qupath.

### CO-Detection by indEXing (CODEX)

Details of the CODEX assay and platform using our selected 21 antibodies conjugated to oligonucleotide barcodes have been previously published^25^. Briefly, 6 cases of NLPHL were incubated with the 21 antibody cocktail. After mounting, and incubation with fluorescent nucleotides and staining, and image acquisition, 4 regions of interest were digitally selected by hematopathologists (S.Y. and Y.N.) to ensure LP cells were present with representative IAPs. Cell types were assessed by co-expression of defining protein markers.

### DNA library preparation and sequencing

Tumor DNA was obtained from 15 cases using laser capture microdissection. Hematoxylin and eosin slides as well as CD20 immunohistochemistry slides were prepared to aid in identification of LP cells. Subsequently, 7 μm thick consecutive sections were cut from formalin-fixed, paraffin embedded blocks on a microtome and were mounted on polyethylene naphthalate membrane slides (Thermo Fisher Scientific LCM0522) with staining performed as previously reported^33^. Cells were dissected using the ArcturusXT LCM system with cutting performed via UV laser and adherence to CapSure HS LCM Caps (Thermo Fisher Scientific LCM0215) using infrared laser. Approximately 200 LP cells were obtained per case. Initially, all efforts were made to isolate individual LP cells with minimal capture of adjacent immune cells, but for later samples to increase successful library preparation, additional adjacent immune cells were also captured to provide additional starting DNA for our precapture sequencing library generation and hybrid capture assay. Uninvolved normal tissue was also dissected for these cases to serve as a source of germline DNA. Cells were digested for 14 h with isolation of DNA using the Arcturus PicoPure DNA Extraction Kit (Thermo Fisher Scientific). DNA was subsequently enzymatically fragmented using KAPA frag (Roche) to a target size of 170 bp.

Cell free DNA was obtained from 6 cases using methods previously described^24^. In brief, plasma was collected in EDTA tubes with isolation of cfDNA using QIAamp Circulating Nucleic Acid Kit (Qiagen). Cellular DNA was isolated from plasma-depleted whole blood using DNeasy Blood and Tissue Kit (Qiagen) with subsequent fragmentation by Kapa frag (Roche).

Precapture libraries were generated as previously described. Barcoded libraries underwent hybrid capture using either 608 kb (SeqCap EZ Choice, Roche) or 772 kb (Discovery Pool, IDT) oligonucleotide panels capturing 151 genes recurrently mutated in Hodgkin and B-cell lymphomas^24^. Captured libraries were sequenced on either Illumina HiSeq4000 or NovaSeq6000 platforms with paired-end 150 bp reads. FASTQ files underwent demultiplexing using 8-bp barcodes and mapping to human reference genome (hg19).

### Genotyping

We used the CAPPseq variant calling platform^24^ to identify somatic tumor mutations in tumor and plasma. Using a reference panel of 16 or more healthy donors for plasma or benign tissue for tumor, we removed background noise. Variant calling was performed with matched germline to remove single nucleotide polymorphisms. Variant calls were filtered to increase specificity: 4 or more variant supporting reads, VAF > 3% for tumor or 0.1% for plasma with duplex support, and enrichment in variant allele frequency from bulk sequencing when available to reduce FFPE and PCR artifact.

### Bulk RNA extraction and sequencing

For each patient, bulk RNA was isolated from 4-6 FFPE slides using RNAstorm FFPE RNA Extraction Kits (Cell Data Sciences). The sequencing libraries were prepared using SMARTer Stranded Total RNA-Seq v2-Pico Input Mammalian Kits (Takara Bio USA, Inc.). Libraries were sequenced on a NovaSeq6000 (Illumina) with 150 base pair paired-end reads. In general each case had a minimum of ∼20 million paired-end reads. The FASTQ files were quasi-aligned to Gencode 27 using Salmon^55^ and expression levels were quantified and summarized as TPM. We limited the genes to only the protein coding genes and normalized the expression to transcripts per million (TPM).

### Deconvolution of bulk RNA-seq data

We leveraged CIBERSORTx^19^ to estimate the proportions of various cell types within bulk RNA from NLPHL tumors. We first created a signature matrix with 23 cell types, incorporating data from a microarray mixture consisting of LP cells and a microarray mixture consisting of 22 human immune cell subsets previously used to construct the LM22 signature matrix (LM22 + LP)^56^. The microarray mixture of LP cells was obtained from GSE12453 which was run on Affymetrix Human Genome U133 Plus 2.0 microarray platform. We performed MAS5 normalization on the data before combining with the already MAS5 normalized lm22 microarray gene expression. For broader cell-type representation, we integrated the TR4 signature matrix^19^. This matrix was derived from bulk RNA-Seq analysis of four distinct cell populations – epithelial, fibroblast, endothelial, and immune cells—isolated through flow sorting. The deconvolution process involved analyzing the bulk RNA sequencing data with both LM22 + LP and TR4 independently. The fractions of all cell types from LM22 + LP were multiplied by the fraction of immune cells from TR4, and were joined by the remaining fractions from TR4 to generate a fraction matrix consisting of 26 cell types, including LP, epithelial, fibroblast, endothelial cells and 22 immune cell types. The fractions of the 25 cell types, not including LP, were pooled into 13 lineages of interest that were seen in high abundance in lymphoma^20^: B cells, dendritic cells, endothelial cells, fibroblasts, mast cells, monocytes and macrophages, NK cells, Neutrophils, Plasma cells, CD4 T cells, CD8 T cells, follicular helper T cells, and regulatory T cells. With the addition of LP cells, the aggregation led to a final fraction matrix containing 14 cell types. Due to seeing an overestimation of LP cells in the signature matrix, we scaled down the samples using genotype information by 16.67 as calculated comparing the LP abundance with twice the variant allele frequency in the bulk genotyping data. Due to this correction and certain other cell types such as epithelial that were removed, we rescaled the entire matrix so that each samples abundance of cell types would sum to 1. To visualize the microenvironment of the different patterns and variant stages, we made stacked barcharts in “ggplot2” and heatmaps of the mean zero, unit variance scaled abundances using “ComplexHeatmap” in R. The ANOVA *p* value across the groups was calculated.

### Discovery of NLPHL cell states

To reveal transcriptionally defined, distinct cell states within cell types, EcoTyper was used to perform NMF along with specialized heuristics. We loaded cell fraction and TPM normalized bulk RNA expression of the patients from discovery cohort as the input. CIBERSORTx HiRes module is used to impute cell type-specific expression profiles from bulk tissue data. The workflow then utilizes NMF to identify transcriptionally-defined cell states within each cell type. The top 1000 genes with highest relative dispersion are used for NMF. Cell types are thrown out if less than 50 genes were imputed in the HiRes module. The cophenetic coefficient for 2–20 clusters was calculated to determine the most stable number of cell states per cell type. The cophenetic coefficient threshold that we selected was 0.95. Posneg transformation was applied to handle non-negativity constraint of NMF. Adaptive false positive index (AFI) filters out spurious cell states caused by negative features from the posneg transformation. Our analysis revealed 34 unique cell states, with each cell type exhibiting 2–4 distinct states. These states were selected for further exploration.

For the UMAP visualizations of each cell type and cell state, we used data generated from CIBERSORTx-purified GEPs for each cell type per sample in the training cohort. Samples were assigned to the dominant cell state based on two criteria: (1) minimum cell state abundance of 33%, and (2) a difference of at least 15% between the most and second most abundant states. The log2-transformed and mean zero unit-variance scaled GEPs were then subjected to dimensionality reduction using the “umap” R package and visualized with “ggplot2.”

### Discovery of NLPHL ecotypes

To delve deeper, we investigated “ecotypes,” or multicellular communities that include distinct cell states that co-occur and co-localize within the microenvironment, potentially representing functionally relevant groupings. Using Jaccard index, how often each pair of states co-occurs is measured, treating each state as a binary value based on its presence in the samples. Statistically significant (*p* > 0.01) overlaps are kept using a hypergeometric test, while non-significant ones are labeled as 0. The Jaccard index matrix of all the pairwise combinations was then subjected to hierarchal clustering and the optimal number of clusters was determined using a threshold of a minimum of 4 cell states per cluster. After assignment, a two-sided *t*-test with unequal variance was calculated to assess for differences on cell state abundances between LPEs relative to abundances of all other cell states in other LPEs with correction for multiple hypothesis testing. The resulting *Q* values are available in Supplementary Tables 25 and 26 and samples with *Q* values less than 0.25 were not assigned. As one final note, the optimal number of EcoTypes is further defined using silhouette width maximization clustering with normalization of EcoType abundances to sum to a 1.0. To illuminate the interconnectedness of ecotypes, we constructed network maps using the “igraph” R package. These maps visually depict cell state relationships, with edges representing similarity as measured by the Jaccard index. LP cell state 3 was visually integrated into LPE3 due to its close proximity to other states within that ecotype.

### Recovery of NLPHL cell states and EcoTypes

For the validation cohort, we followed EcoTyper’s recovery framework to map and quantify previously identified NLPHL cell states and ecotypes within a validation cohort consisting of 62 patients. Utilizing the cell type specific matrix from the prebuilt NMF model, we determined the mixture coefficient matrix for each cell type. Each cell state from the validation cohort gets incorporated as a weight. This adaptable framework accommodates diverse profiling methods, including bulk RNA-Seq, microarrays, and scRNA-Seq. After cell states were quantitated, the NLPHL ecotype abundance was determined as described in discovery of NLPHL ecotypes.

### Single nucleus RNA sequencing

Nuclei from fresh frozen NLPHL lymph node excision specimens were isolated and prepared for sequencing using the Chromium 10X assay (3’ v3.1 by 10X genomics). Initial steps involved sample demultiplexing, read alignment to the GRCh38 human reference genome, and the creation of feature-barcode matrices using CellRanger v6.0.0 (10x Genomics). Subsequently, Seurat v4.178^57,58^ was employed for the analysis and annotation of our single cell cohort. Cells meeting the criteria of ≥10% of reads mapped to mitochondrial genes and having ≤200 or ≥5000 expressed genes were excluded from the analysis. After normalizing and finding variable features, we then proceeded to find integration anchors as input to Seurat’s IntegrateData() function which allowed us to combine the samples together. We preprocessed the data by scaling it, before performing principal component analysis using the top 2000 most variable genes to capture major data variance. Subsequently, we employed the FindNeighbors() function in Seurat to identify k-nearest neighbors for each cell within the high-dimensional space defined by the top 30 PCs, chosen based on the jackstraw procedure and visualized with an elbow plot. Utilizing the k-nearest neighbor information, we constructed a shared nearest neighbor graph using the FindClusters() function within Seurat. Cluster identification was achieved with a resolution parameter of 1.25. Notably, similar clustering patterns were observed with different PC selections and resolution values, confirming the robustness of the identified cell groupings.

### Annotation of snRNA-Seq

For the annotation of cell types within our dataset, we looked at the top 50 marker genes within each cluster and labeled each cluster according to previously reported information on the marker genes. We utilized celltypist version 1.6.0^59^ to further validate our annotations. We used the prebuilt “Immune_All_Low” model which was constructed from 20 tissues and 18 studies of immune sub-populations. The most common cells identified by celltypist for each cluster was used to validate our cluster assignments. To understand the landscape of copy number alterations within B cells, plasma cells, and LP cells, we employed inferCNV^44^ version 1.16.0 with the other immune cell types in our single cell samples as our normal cells. We employed InferCNV three separate times, with analyses performed on combined samples once and on each single nucleus sample separately. The parameters were left as default, but we set HMM = “true”, cutoff = 0.1, and tumor_subcluster_partition_method = “qnorm”. To interpret copy number calls from inferCNV, we replaced “6” (gain > 2) with “5” and shifted all values by 3 to make “0” the neutral reference point. For a global assessment of CNAs, we calculated the sum of absolute gene-level changes per cell. We then compared these summed values across cell types using the Wilcoxon rank-sum test to identify significant differences. Specifically, for CD274 (*PD-L1*), we analyzed copy number changes without taking absolute values to distinguish amplifications and deletions, followed by another Wilcoxon rank-sum test for significance.

### Recovery of EcoTyper cell states in snRNA-Seq

The annotated single-cell transcriptomes were mapped to EcoTyper cell states using the single cell recovery module. The statistical significance of cell state recovery was assessed using permutation testing, as previously described^60^ to generate a z-score indicating statistical confidence.

### Benchmarking of CIBERSORTx method

We calculated the Pearson correlation coefficient and derived *P* values for the abundances deconvolved using the CIBERSORTx signature matrix, which was based on microarray data, and compared them to the standard single-cell measured abundance profiles of overlapping cases from CODEX. Additionally, we constructed signature matrices using other established platforms, including MuSiC and DLWS, along with CIBERSORTx employing a snRNA-seq derived signature matrix. Both DLWS version 0.1.0 and MuSiC version 1.0.0 were applied to our single nucleus RNA-seq data, utilizing default parameters. CIBERSORTx was rerun with snRNA-seq data to determine cell type proportions in our bulk discovery cohort. Subsequently, we calculated the Pearson correlation coefficients and obtained *P* values for each cell type enumeration from the different methods, comparing them against the overlapping cases in CODEX.

### Spatial transcriptomics sequencing

Fresh lymph node excision specimens were harvested and frozen in optimal cutting temperature compound. They were cryosectioned at −20 °C by the Stanford Genomics Shared Resource. Several iterations to obtain the optimal permeabilization time was performed using 10 μm sections with the Visium Spatial Tissue Optimization Slide and Reagents Kit (10X genomics). Subsequently, sequencing libraries were generated using the Visium Spatial Gene Expression Slide and Reagent Kit (10X Genomics). The library was sequenced on a NovaSeq6000 (Illumina). For the two regions from sample LP152, Space Ranger version 2.0.0 was employed, utilizing the count function for tissue fiducial detection, alignment to the GRCh38 human reference genome, and the generation of processed data files, including feature-barcode matrices. For samples LP16, LP17, LP18, and LP20, Space Ranger version 3.0.0 was used. The count function was again utilized to generated spatial gene expression profiles from the raw sequencing data, incorporating CytAssist images and performing read alignment to the GRCh38 human reference genome.

### Recovery of cell states and EcoTypes in spatial transcriptomics

We used the feature-barcode matrices to estimate cell state and ecotype abundance for each spatially-barcoded spot. We following previously established procedures^60^ which, in summary, starts by estimating the fractional abundance of NLPHL cell states for each spot. Subsequently, the most prevalent cell state for each cell type within a spot was designated as 1, with the others set to 0. CIBERSORTx was used to determine cell type abundances for each spot so that we could normalize each cell state abundance by its the parent cell type fraction through multiplication. Abundances were then scaled such that the 99th percentile within the cell type was set to 1. For each ecotype, we averaged the individual state abundances within that ecotype of each spot to find its abundance. Moran’s I^61^, a test of spatial aggregation, was utilized to investigate whether specific ecotypes tended to co-localize within the tissue. Euclidean distance was calculated to determine the distance between spots.

### Immunohistochemistry for HLA-I and HLA-II

Tissue microarrays were constructed for all cases with remaining tissue after confirming diagnosis and performing genotyping and RNA-seq. Antibodies for B2M, HLA-I and HLA-II were used as previously reported^43^. Protein expression for LP cells was quantified using QuPath.

### Immune repertoire

We used Trust4 (version 1.0.8) with HG38 B cell receptor (BCR) and T cell receptor (TCR) fasta file which contains coordinate and sequence of V/D/J/C genes as a reference, along with the IMGT database V/D/J/C gene reference file. We separated the TCR and BCR sequences and calculated the Shannon entropy for each sample, using the frequency of each TCR or BCR sequence. We normalized the input frequencies by dividing each count by the total number of TCR or BCR sequences in the respective sample. These entropies were then analyzed based on ecotype and relapse status. To incorporate all the samples, we used the highest abundance to assign LP ecotype, although the results stayed consistent when using ecotyper assigned LP ecotypes. We tested significance using a two-sided Wilcoxon rank-sum *t*-test. To explore CDR3 motifs in CD4 and CD8 T cells, the T cell receptor sequences from TRUST4 was input into GLIPH2 version 0.01. Using the GLIPH2 output, we performed a Wilcoxon rank-sum *t*-test comparing the Shannon entropies of the CDR3 motif clusters per sample in T cell receptors against Ecotypes and relapse status. To investigate potential past bacterial exposure, we compared the BCR sequences identified in our samples against curated BCR sequences extracted from the Thurner et al. paper. We used NCBI BLAST version 2.11.0 and required a stringent *e* value threshold of 1.0e-5 to find significant matches.

### Statistical analyses

Clinical follow-up time was calculated using the reverse Kaplan-Meier method. The primary clinical outcome was FFP with progression measured from date of initial diagnosis to date of NLPHL recurrence or transformation to large B-cell lymphoma. FFP was chosen as there were very few deaths observed in either the training or validation cohorts representing minimal competing risk. Survival curves were generated using survfit module from “survival”, using a Kaplan-Meier method. To calculate hazard ratios and perform additional analyses, we performed a stratified Cox regression accounting for management type using the “crrc” R module. The Spearman correlation coefficient was used to measure the correlation between variables/cohorts. Unless indicated otherwise, Mann-Whitney U was used to calculate *P* values for experimental data. All *P* values were two-tailed and considered significant at alpha <0.05. We utilized R version 4.1.2, executed in both R Server and R console environments, as well as Python version 3.8.8.

### Reporting summary

Further information on research design is available in the Nature Portfolio Reporting Summary linked to this article.

## Supplementary information

Supplementary Information

Transparent Peer Review file

Reporting Summary

Description of Additional Supplementary Files

Supplementary Data 1-26

## Source data

Source Data

## Data Availability

Deidentified patient summary data, gene expression data from bulk RNA-seq (expressed as TPM), and genotyping results are available in the Supplementary Data. Raw expression counts for the single nucleus RNA-seq after mapping and quantification by Cell Ranger is available in the Supplementary Data. Additionally, raw expression counts for the Visium data after mapping and quantification by Space Ranger are available in the Source Data file. Due to the nature of international protected health information, data sharing and tissue sharing contract agreements, raw sequencing files are not available for download due to risk of potential identification of individuals using single nucleotide polymorphisms and other genomic markers. Requests for raw sequencing files are subject to individual institutional data use agreements as patient consent to supply this information publicly outside of a secure data use agreement was not obtained. Supplementary figures are available in the Supplementary Information file. Source data are provided with this paper.

## Code availability

The code used for CIBERSORTx, EcoTyper, TRUST4, and GLIPH2 is publicly available. The custom NLPHL signature matrix and discovery EcoTyper model is available at https://github.com/msbinkley/LPEcoTyper/
