# Supplementary material for: Distinct cell state ecosystems for nodular lymphocyte-predominant Hodgkin lymphoma
Source: Nat Commun. 2025 Sep 26;16:8473. doi: 10.1038/s41467-025-63339-9 (PMC12475200; doi:10.1038/s41467-025-63339-9)
Supplement: Supplementary file 1 — Supplementary Information [file 41467_2025_63339_MOESM1_ESM.pdf]

Supplementary Figure 1.

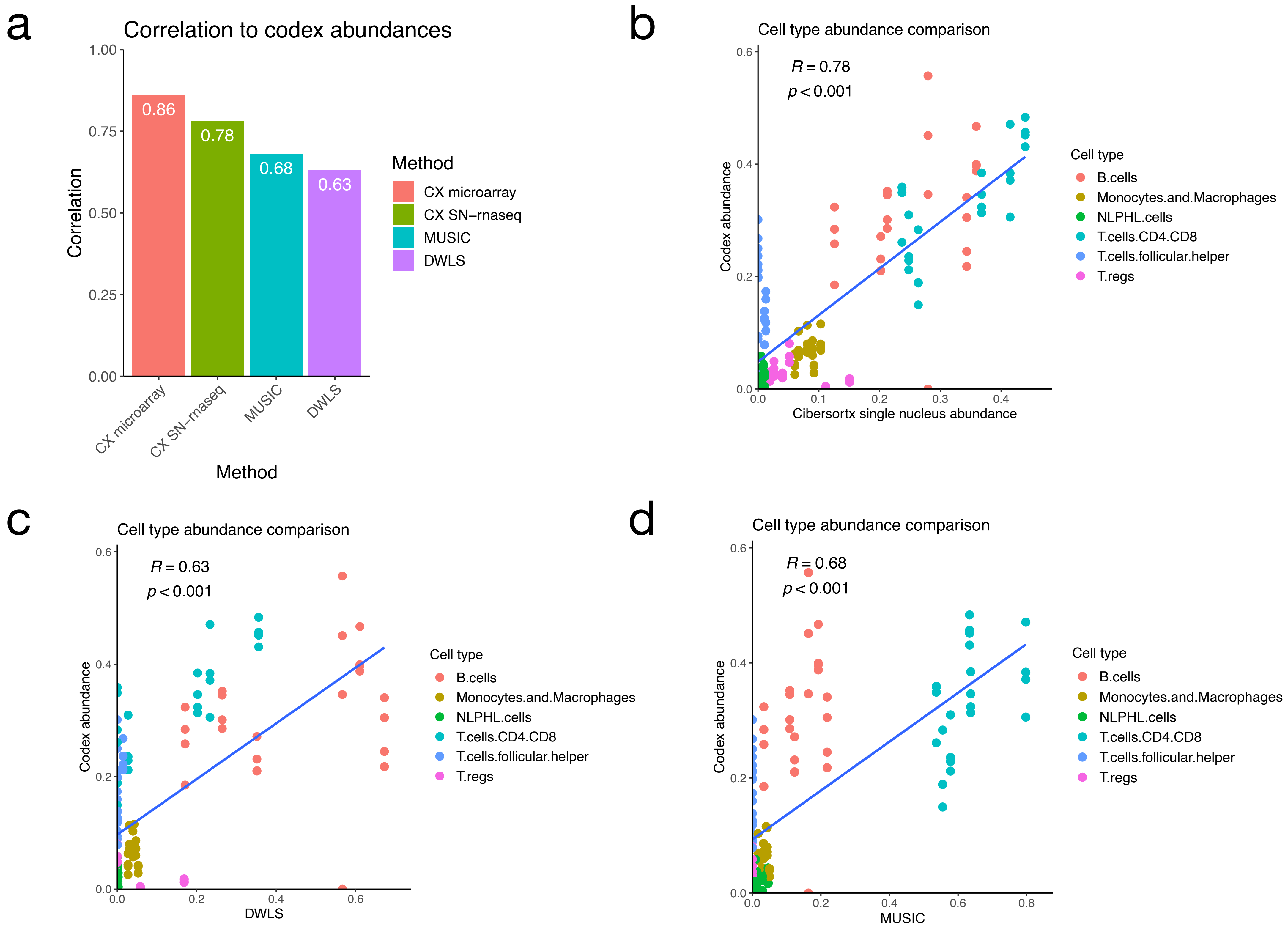

Supplementary Fig. 1. a, Bar chart summarizes the correlation between 4 digital bulk RNA-seq deconvolution methods and CODEX derived cell abundances. Correlation plots compare cell abundances obtained for 6 cases with 4 regions of interest selected per case using CODEX versus values obtained using CIBERSORTx with a snRNA-seq signature matrix (b), DWLS (c), and MuSiC (d). For panels a,b,c,d, the Pearson correlation was used to determine linear fit and t-test was used to determine the statistical significance of a correlation coefficient)

Supplementary Figure 2.

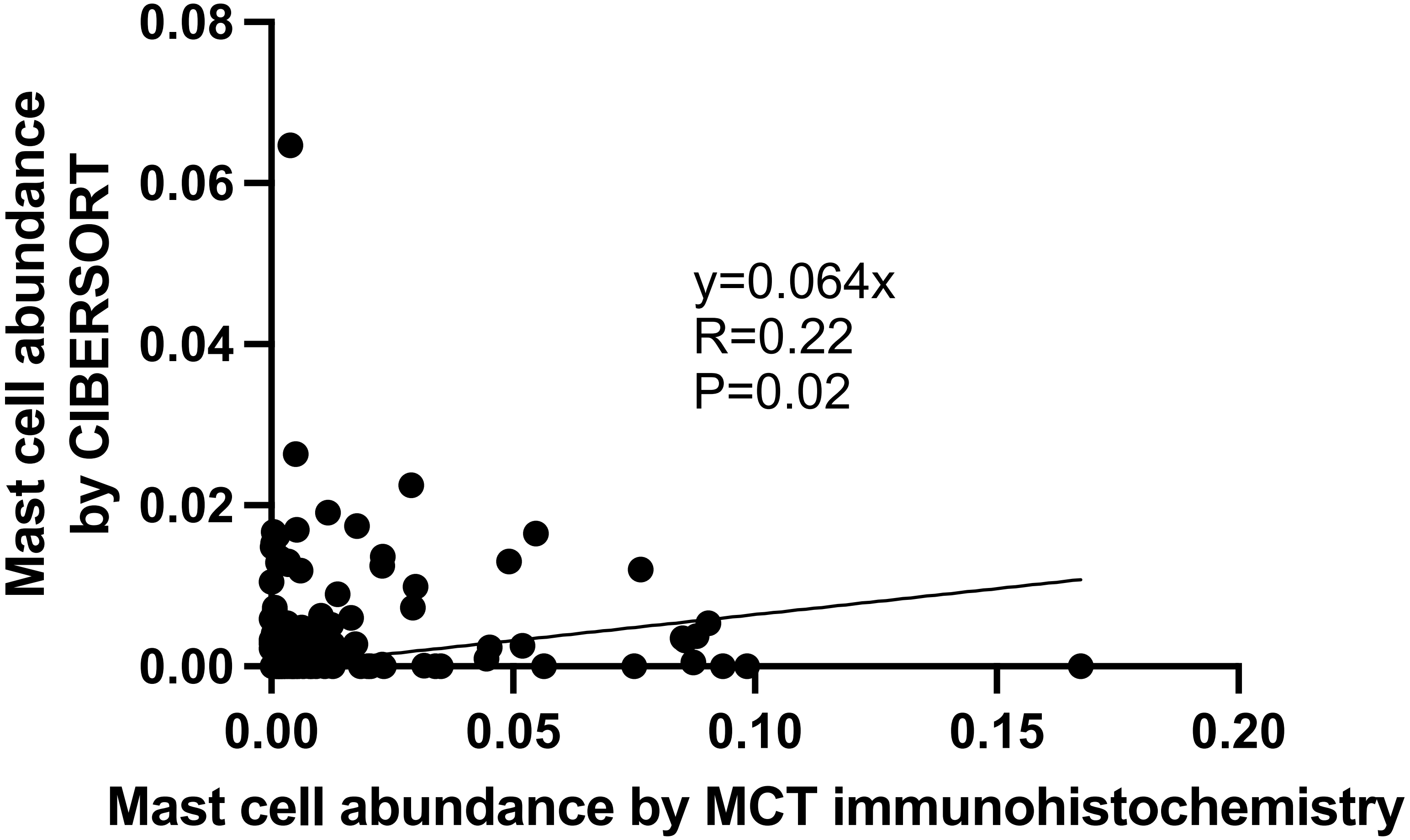

Supplementary Fig. 2. Correlation between mast cell abundance obtained via CIBERSORTx compared to abundance measured by QuPath using immunohistochemistry for MCT. Pearson correlation is shown along with two sided P-value derived from t-test.

Supplementary Figure 3.

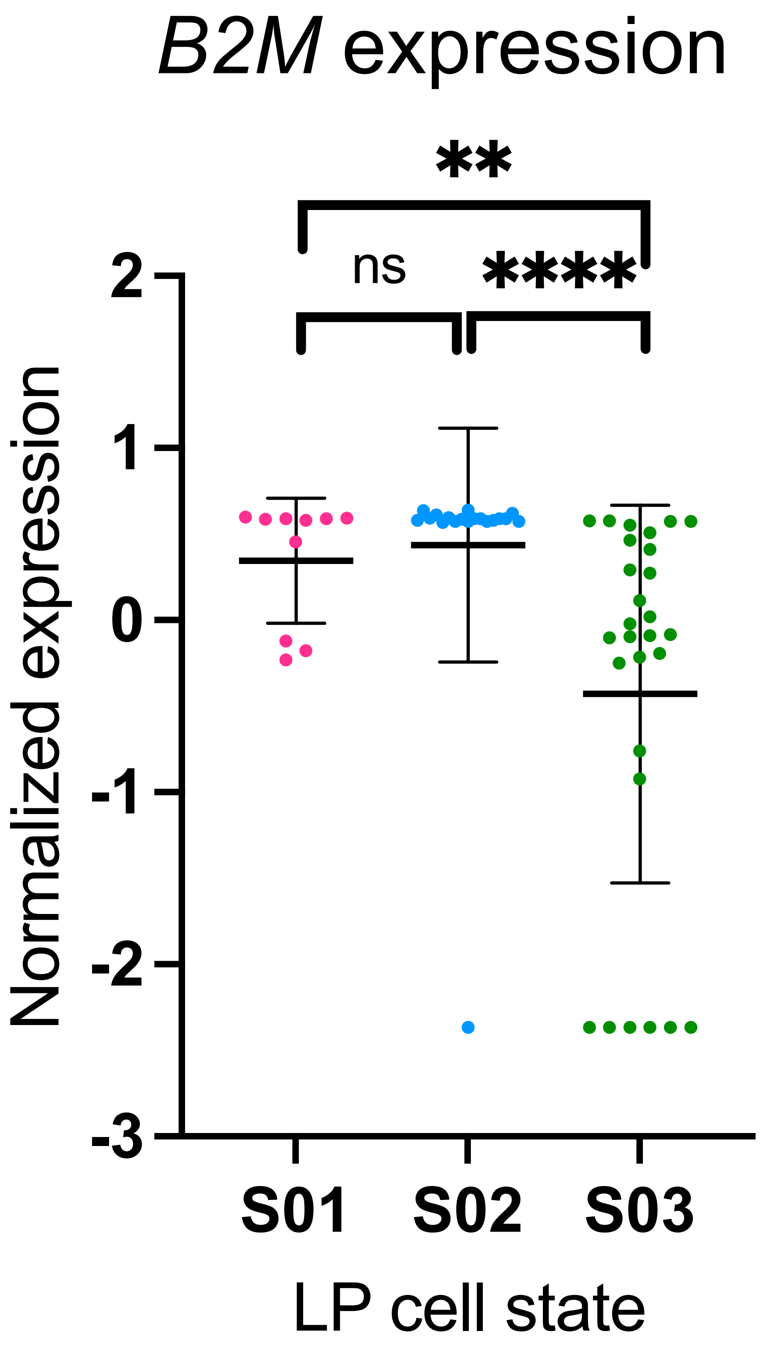

Supplementary Fig. 3. Scatter bar plot shows B2M expression is significantly higher for LPS01 and LPS02 versus LPS03. The median along with IQR and P-values which were derived from two-sided Wilcoxon rank-sum t-test are shown.

Supplementary Figure 4.

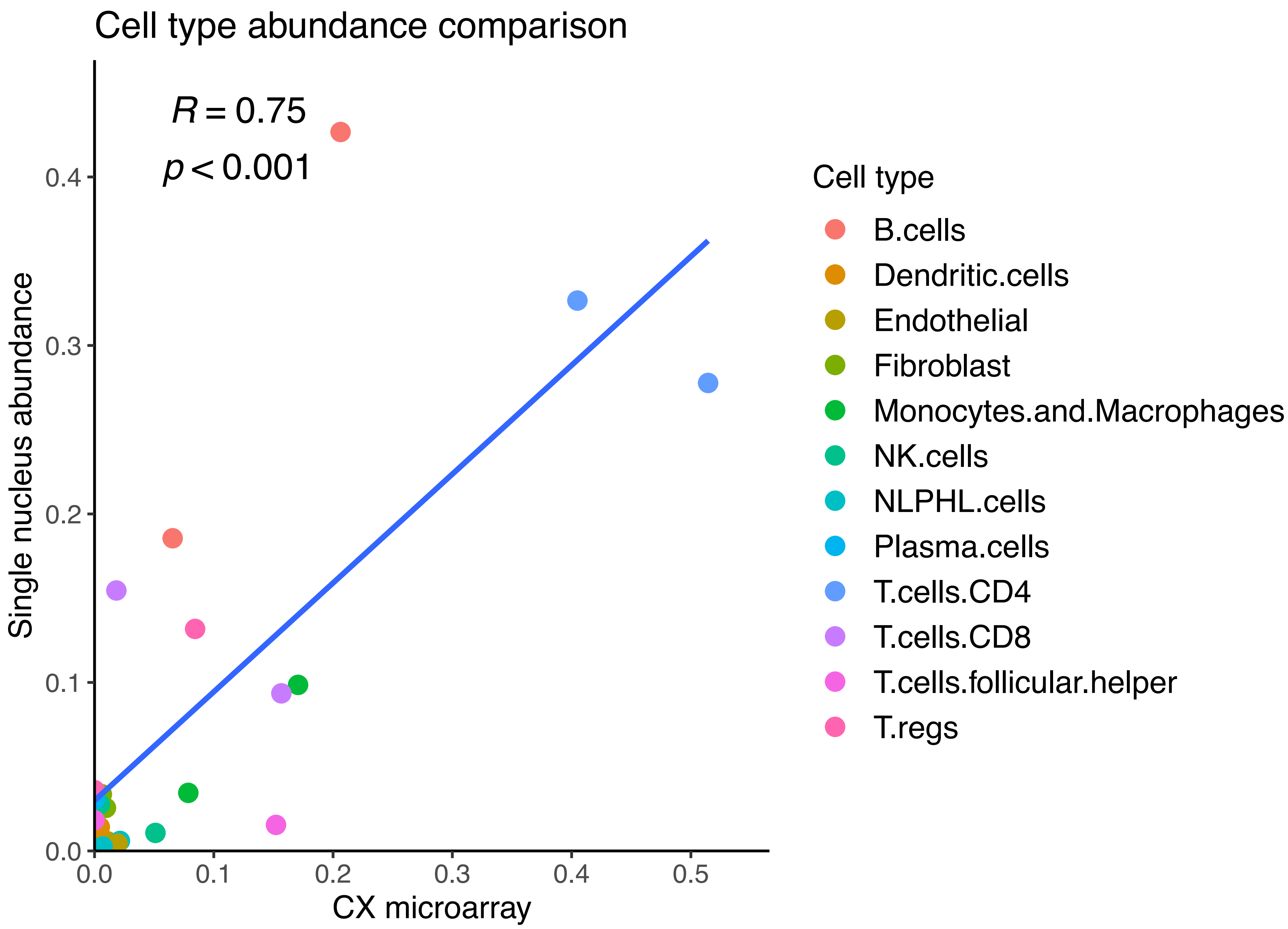

Supplementary Fig. 4. Scatter plot shows significant correlation between cell abundances obtained for 2 NLPHL samples profiled by CIBERSORTx with bulk digital deconvolution versus snRNA-seq. Pearson correlation is shown along with two sided P-value derived from t-test.

Supplementary Figure 5.

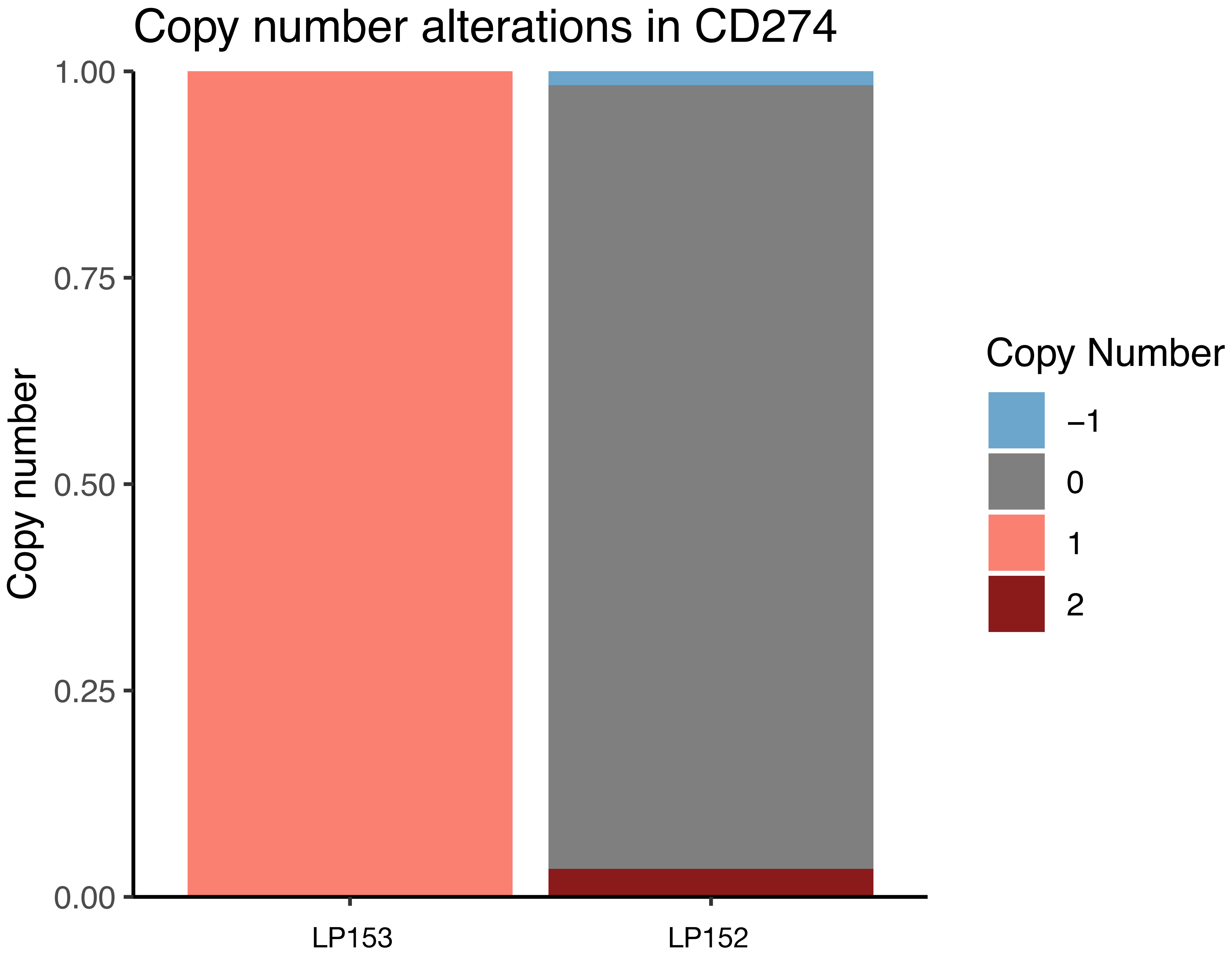

Supplementary Fig. 5. Abundance of CD274 copy amplifications for LP152 as measured from snRNA-seq data using inferCNV.

Supplementary Figure 6.

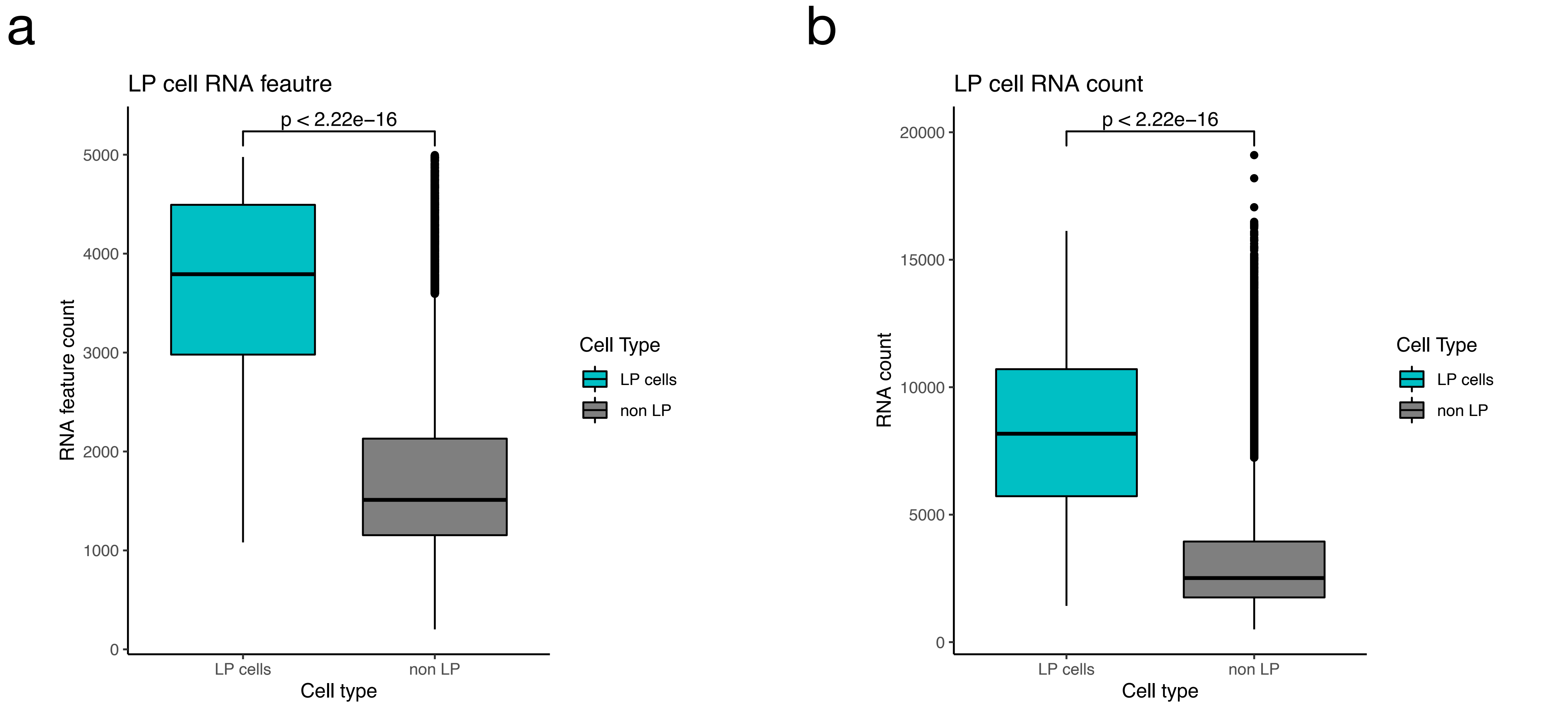

Supplementary Fig. 6. Box and whisker plots show LP cells had significantly higher RNA feature count (unique genes) in panel a and n count (total number of RNA molecules per cell) in panel b relative to other cell types. For panels a and b, we display the median value and IQR for the boxplot with whiskers determined by Tukey method. The P-values are obtained from two-sided Wilcoxon rank-sum tests.

Supplementary Figure 7.

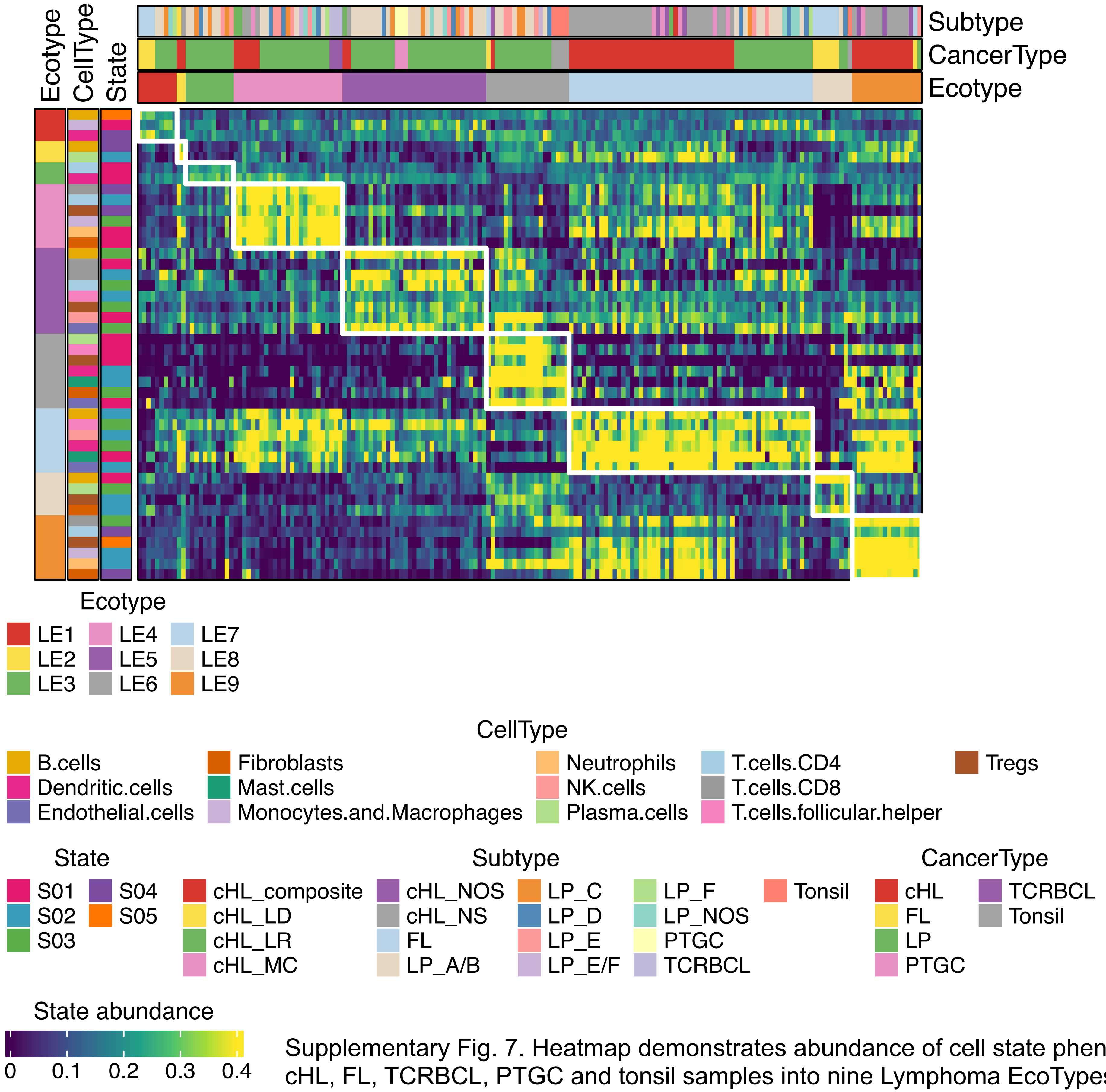

Supplementary Fig. 7. Heatmap demonstrates abundance of cell state phenotypes and clustering of NLPHL, cHL, FL, TCRBCL, PTGC and tonsil samples into nine Lymphoma EcoTypes.

Supplementary Figure 8.

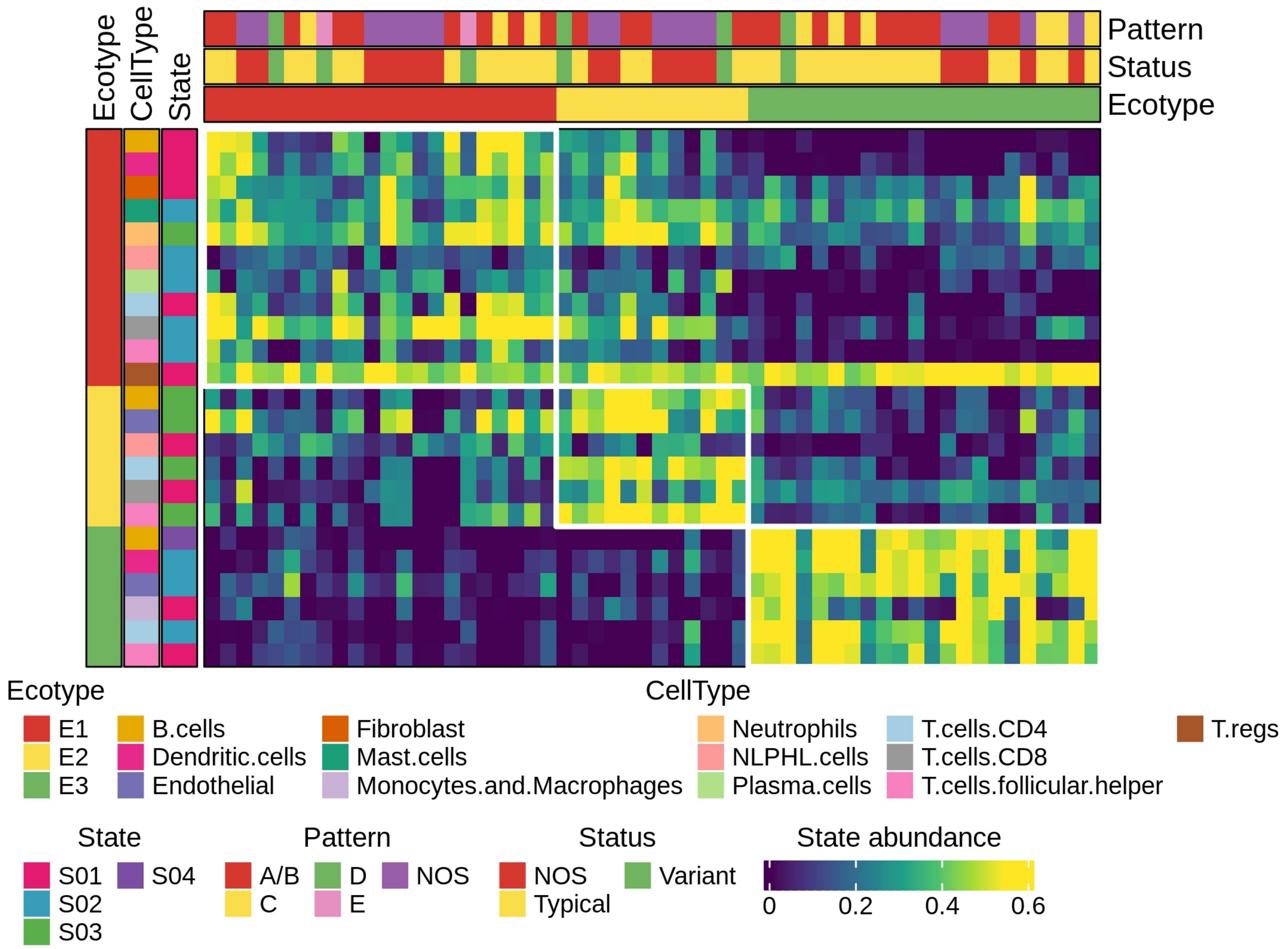

Supplementary Fig. 8. Heatmap demonstrates abundance of cell state phenotypes and clustering of the validation cohort into three LPEs

Supplementary Figure 9.

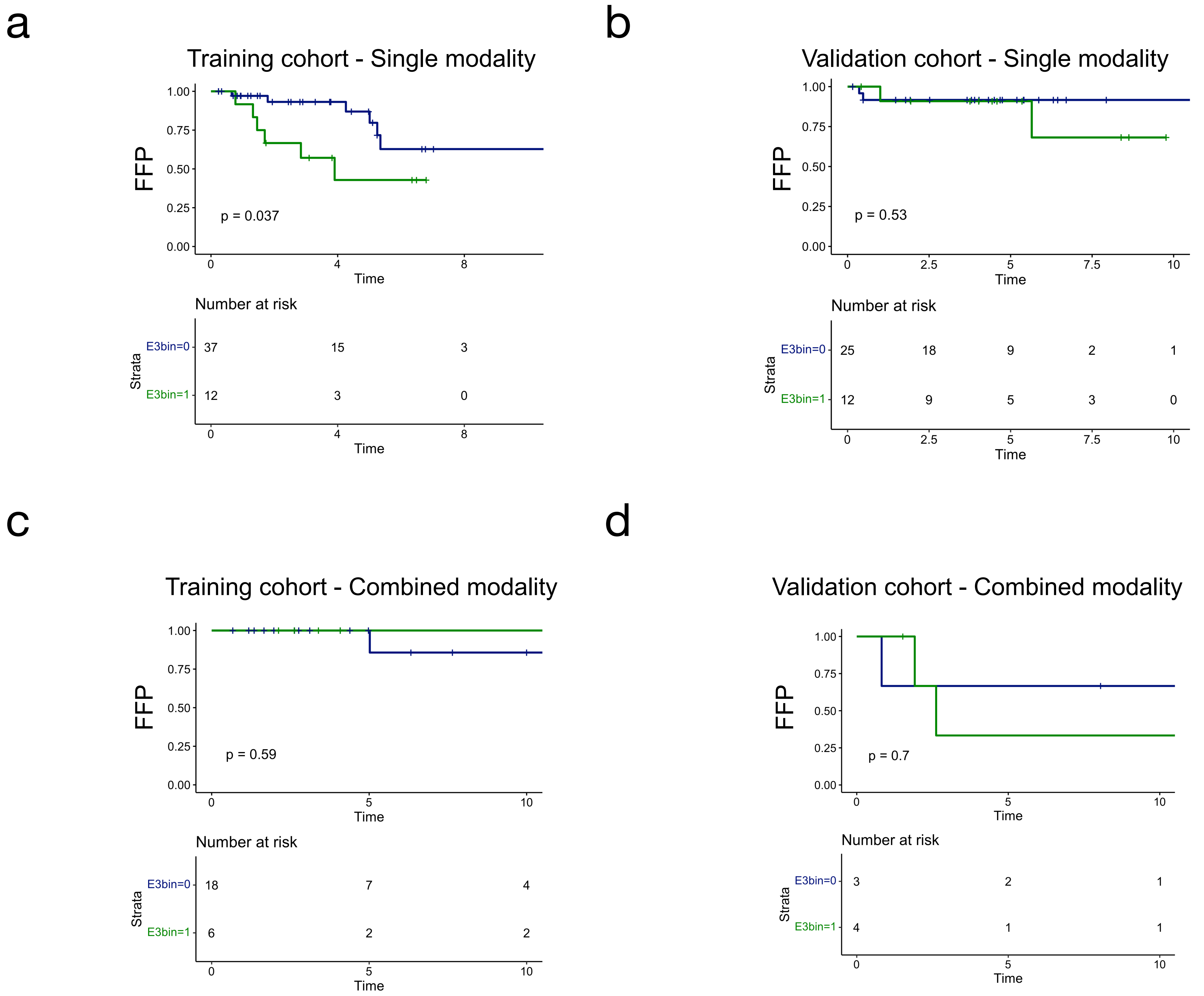

Supplementary Fig. 9. Kaplan-Meier curves show patients who received single modality with LPE3 versus LPE1-2 for the training (a) and validation (b) cohorts. k, Kaplan-Meier curves show patients who received combined modality with LPE3 versus LPE1-2 for the training (c) and validation (d) cohorts.

Supplementary Figure 10.

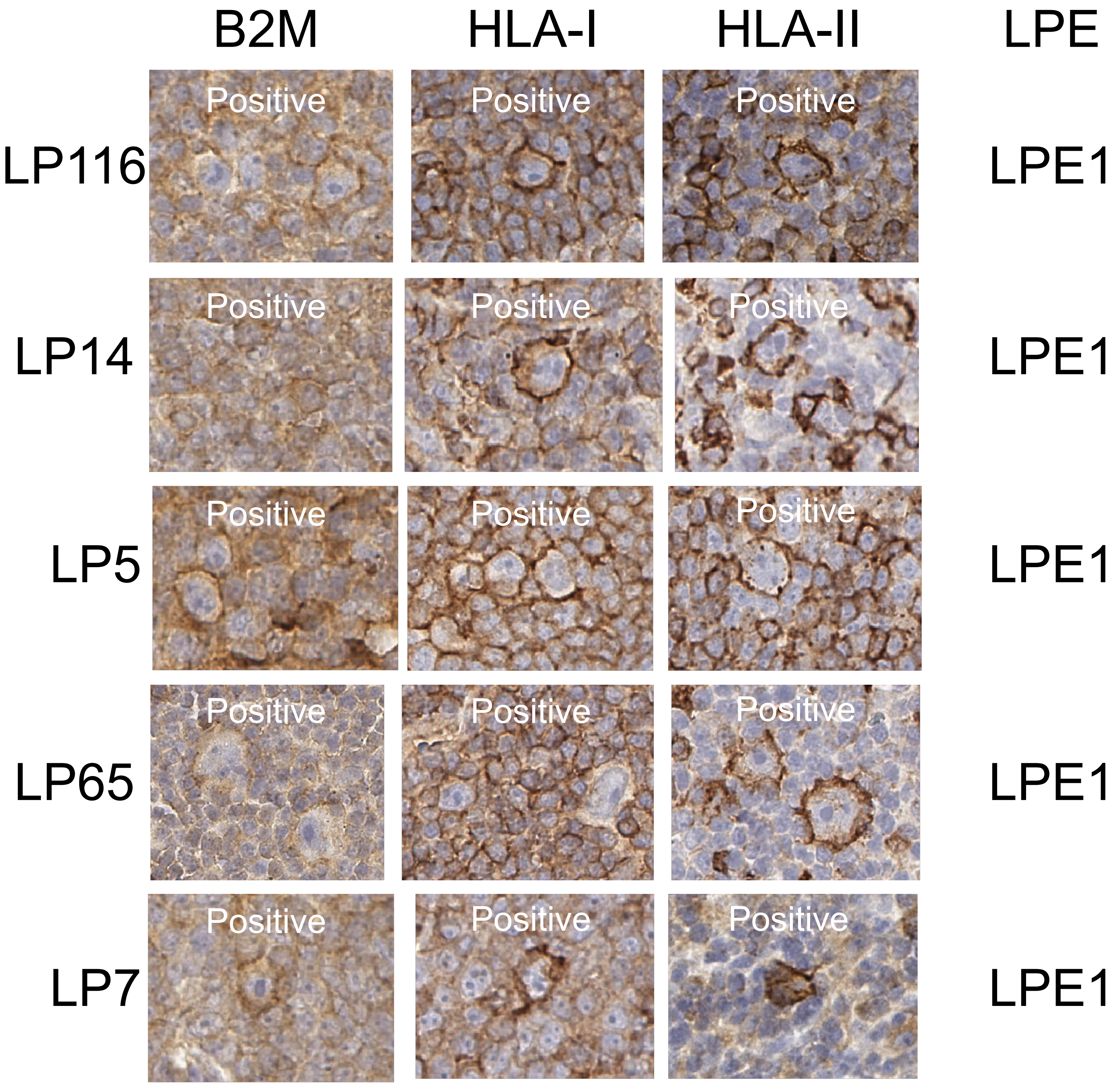

Supplementary Fig. 10. Representative examples of B2M positive NLPHL samples with additional staining for HLA-I and HLA-II with LPE assignment and data in Supplementary Table 24.

Supplementary Figure 11.

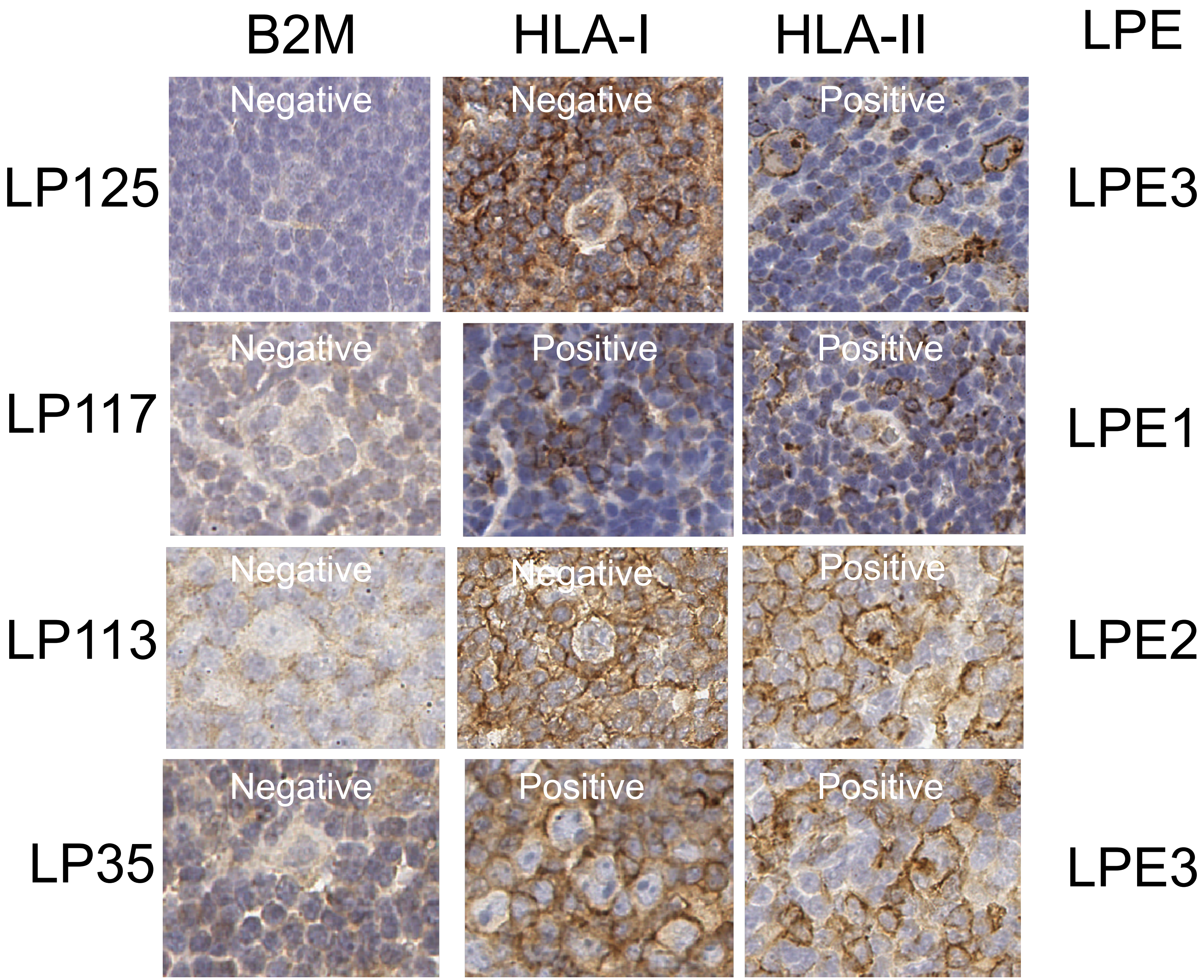

Supplementary Fig. 11. Representative examples of B2M negative NLPHL samples with additional staining for HLA-I and HLA-II with LPE assignment and data in Supplementary Table 24.
