# Supplementary material for: Distinct cell state ecosystems for nodular lymphocyte-predominant Hodgkin lymphoma
Source: Nat Commun. 2025 Sep 26;16:8473. doi: 10.1038/s41467-025-63339-9 (PMC12475200; doi:10.1038/s41467-025-63339-9)
Supplement: Supplementary file 3 — Reporting Summary [file 41467_2025_63339_MOESM3_ESM.pdf]

nature portfolio

Corresponding author(s): Michael Sargent Binkley

Last updated by author(s): Feb 20, 2024

Reporting Summary

Nature Portfolio wishes to improve the reproducibility of the work that we publish. This form provides structure for consistency and transparency in reporting. For further information on Nature Portfolio policies, see our Editorial Policies and the Editorial Policy Checklist.

Statistics

For all statistical analyses, confirm that the following items are present in the figure legend, table legend, main text, or Methods section.

n/a	Confirmed	
☐	☒	The exact sample size (*n*) for each experimental group/condition, given as a discrete number and unit of measurement
☐	☒	A statement on whether measurements were taken from distinct samples or whether the same sample was measured repeatedly
☐	☒	The statistical test(s) used AND whether they are one- or two-sided<br>*Only common tests should be described solely by name; describe more complex techniques in the Methods section.*
☐	☒	A description of all covariates tested
☐	☒	A description of any assumptions or corrections, such as tests of normality and adjustment for multiple comparisons
☐	☒	A full description of the statistical parameters including central tendency (e.g. means) or other basic estimates (e.g. regression coefficient) AND variation (e.g. standard deviation) or associated estimates of uncertainty (e.g. confidence intervals)
☐	☒	For null hypothesis testing, the test statistic (e.g. *F*, *t*, *r*) with confidence intervals, effect sizes, degrees of freedom and *P* value noted<br>*Give P values as exact values whenever suitable.*
☒	☐	For Bayesian analysis, information on the choice of priors and Markov chain Monte Carlo settings
☐	☒	For hierarchical and complex designs, identification of the appropriate level for tests and full reporting of outcomes
☐	☒	Estimates of effect sizes (e.g. Cohen's *d*, Pearson's *r*), indicating how they were calculated

Our web collection on statistics for biologists contains articles on many of the points above.

Software and code

Policy information about availability of computer code

Data collection	For all gene expression data and nucleotide base calling, the specific softwares and version numbers used for quantification are provided.
Data analysis	For all gene expression normalization, and filtering of variant calling, the specific softwares, version numbers, and relevant prior publications establishing methods have been provided.

For manuscripts utilizing custom algorithms or software that are central to the research but not yet described in published literature, software must be made available to editors and reviewers. We strongly encourage code deposition in a community repository (e.g. GitHub). See the Nature Portfolio guidelines for submitting code & software for further information.

Data

Policy information about availability of data

All manuscripts must include a data availability statement. This statement should provide the following information, where applicable:

- Accession codes, unique identifiers, or web links for publicly available datasets
- A description of any restrictions on data availability
- For clinical datasets or third party data, please ensure that the statement adheres to our policy

Deidentified patient summary data, gene expression data, and genotyping results are available in the Supplementary Data. Due to the nature of protected health information, data sharing and tissue sharing agreements, raw sequencing files are not available for download.

Research involving human participants, their data, or biological material

Policy information about studies with human participants or human data. See also policy information about sex, gender (identity/presentation), and sexual orientation and race, ethnicity and racism.

Reporting on sex and gender	Gender was provided based on self reporting by the patient care providers who submitted clinical data and pathologic tissue.
Reporting on race, ethnicity, or other socially relevant groupings	Race and ethnicity was not reported by the data contributers for the study.
Population characteristics	The patient demographics including age, gender, clinical stage, and additional information is included in our supplementary data. We also describe the summary results in the Main Text of the manuscript.
Recruitment	This was a retrospective study with omission of patient consent as deemed appropriate by the individual review boards of the participating centers. As such, data availability reflects the protected nature of some elements of the data used in the study. All other data is available and included in our Supplementary Tables.
Ethics oversight	Each individual hospital had institutional review board approval to conduct this retrospective study.

Note that full information on the approval of the study protocol must also be provided in the manuscript.

Field-specific reporting

Please select the one below that is the best fit for your research. If you are not sure, read the appropriate sections before making your selection.

☒ Life sciences ☐ Behavioural & social sciences ☐ Ecological, evolutionary & environmental sciences

For a reference copy of the document with all sections, see nature.com/documents/nr-reporting-summary-flat.pdf

Life sciences study design

All studies must disclose on these points even when the disclosure is negative.

Sample size	Based on the published EcoTyper software sample size recommendations (3 samples per cell type) and the number of cell types included in our study, the minimum number of samples to be included in the training and validation cohorts was 42 each. Our sample sizes exceed this estimate in both the training and validation cohorts.
Data exclusions	No data was excluded from submitted cases and tissue samples. Some tissue samples did not have paired clinical data, but all samples were used for generation of our EcoTyper models.
Replication	We collected cases and tissue for an independent validation cohort as described in the Main Text.
Randomization	This was a retrospective study that did not include randomization.
Blinding	This was a retrospective study that did not include blinding.

Reporting for specific materials, systems and methods

We require information from authors about some types of materials, experimental systems and methods used in many studies. Here, indicate whether each material, system or method listed is relevant to your study. If you are not sure if a list item applies to your research, read the appropriate section before selecting a response.

Materials & experimental systems

n/a	Involved in the study	
☐	☒	Antibodies
☒	☐	Eukaryotic cell lines
☒	☐	Palaeontology and archaeology
☒	☐	Animals and other organisms
☐	☒	Clinical data
☒	☐	Dual use research of concern
☒	☐	Plants

Methods

n/a	Involved in the study	
☒	☐	ChIP-seq
☒	☐	Flow cytometry
☒	☐	MRI-based neuroimaging

Antibodies

Antibodies used	MHC class I (Abcam, EMR8-5, 1:6,000), and MHC class II (Dako, CR3/43 M0775, 1:750)
Validation	This was performed according to published methods (reference 40).

Clinical data

Policy information about clinical studies

All manuscripts should comply with the ICMJE guidelines for publication of clinical research and a completed CONSORT checklist must be included with all submissions.

Clinical trial registration	n/a
Study protocol	n/a
Data collection	n/a retrospective study.
Outcomes	n/a retrospective study.

Plants

Seed stocks	n/a
Novel plant genotypes	n/a
Authentication	n/a
