# Supplementary material for: Distinct cell state ecosystems for nodular lymphocyte-predominant Hodgkin lymphoma
Source: Nat Commun. 2025 Sep 26;16:8473. doi: 10.1038/s41467-025-63339-9 (PMC12475200; doi:10.1038/s41467-025-63339-9)
Supplement: Supplementary file 4 — Description of Additional Supplementary Files [file 41467_2025_63339_MOESM4_ESM.pdf]

Description of Additional Supplementary Files

Supplementary Data 1
Description: Baseline characteristics of training cohort.

Supplementary Data 2
Description: Baseline characteristics of validation cohort.

Supplementary Data 3
Description: Summary statistics for training and validation cohorts.

Supplementary Data 4
Description: NLPHL specific signature matrix.

Supplementary Data 5
Description: CIBERSORT results for tonsil.

Supplementary Data 6
Description: CIBERSORT results for whole blood.

Supplementary Data 7
Description: CIBERSORT results for progressive transformation of germinal centers (PTGC).

Supplementary Data 8
Description: TPM gene expression matrix for training cohort.

Supplementary Data 9
Description: TPM gene expression matrix for validation cohort.

Supplementary Data 10
Description: Genotyping results after filtering obtained from sequencing DNA isolated from tissue or plasma.

Supplementary Data 11
Description: Cell state defining genes for B-cells.

Supplementary Data 12
Description: Cell state defining genes for Dendritic cells.

Supplementary Data 13
Description: Cell state defining genes for Endothelial cells.

Supplementary Data 14
Description: Cell state defining genes for fibroblasts.

Supplementary Data 15.
Description: Cell state defining genes for LP cells.

Supplementary Data 16
Description: Cell state defining genes for Mast cells.

Supplementary Data 17
Description: Cell state defining genes for Macrophages.

Supplementary Data 18
Description: Cell state defining genes for Neutrophils.

Supplementary Data 19
Description: Cell state defining genes for Plasma cells.

Supplementary Data 20
Description: Cell state defining genes for T-cells CD4.

Supplementary Data 21
Description: Cell state defining genes for T-cells CD8.

Supplementary Data 22
Description: Cell state defining genes for Tfh.

Supplementary Data 23
Description: Cell state defining genes for Tregs.

Supplementary Data 24
Description: Immunostain results for B2M, HLA-I, and HLA-II.

Supplementary Data 25
Description: Q-values for EcoTyper Training Cohort.

Supplementary Data 26
Description: Q-values for EcoTyper Validation Cohort.
